# Impact of the Method of Delivering Electronic Health Behavior Change Interventions in Survivors of Cancer on Engagement, Health Behaviors, and Health Outcomes: Systematic Review and Meta-Analysis

**DOI:** 10.2196/16112

**Published:** 2020-06-23

**Authors:** Kate Furness, Mitchell N Sarkies, Catherine E Huggins, Daniel Croagh, Terry P Haines

**Affiliations:** 1 Nutrition and Dietetics Monash Medical Centre Monash Health Melbourne Australia; 2 School of Primary and Allied Health Care Faculty of Medicine, Nursing and Health Sciences Monash University Melbourne Australia; 3 Department of Physiotherapy, School of Primary and Allied Health Care Faculty of Medicine, Nursing and Health Sciences Monash University Melbourne Australia; 4 School of Public Health Faculty of Health Sciences Curtin University Perth Australia; 5 Centre for Healthcare Resilience and Implementation Science Australian Institute of Health Innovation Faculty of Medicine, Health and Human Sciences Macquarie University Sydney Australia; 6 Department of Nutrition, Dietetics and Food, School of Clinical Sciences Faculty of Medicine, Nursing and Health Sciences Monash University Melbourne Australia; 7 Upper Gastrointestinal and Hepatobiliary Surgery Monash Medical Centre Monash Health Melbourne Australia; 8 Department of Surgery, School of Clinical Sciences Faculty of Medicine, Nursing and Health Sciences Monash University Melbourne Australia

**Keywords:** eHealth, mHealth, behavior, neoplasm, mobile phones

## Abstract

**Background:**

Increased accessibility to the internet and mobile devices has seen a rapid expansion in electronic health (eHealth) behavior change interventions delivered to patients with cancer and survivors using synchronous, asynchronous, and combined delivery methods. Characterizing effective delivery methods of eHealth interventions is required to enable improved design and implementation of evidence-based health behavior change interventions.

**Objective:**

This study aims to systematically review the literature and synthesize evidence on the success of eHealth behavior change interventions in patients with cancer and survivors delivered by synchronous, asynchronous, or combined methods compared with a control group. Engagement with the intervention, behavior change, and health outcomes, including quality of life, fatigue, depression, and anxiety, were examined.

**Methods:**

A search of Scopus, Ovid MEDLINE, Excerpta Medica dataBASE, Cumulative Index to Nursing and Allied Health Literature Plus, PsycINFO, Cochrane CENTRAL, and PubMed was conducted for studies published between March 2007 and March 2019. We looked for randomized controlled trials (RCTs) examining interventions delivered to adult cancer survivors via eHealth methods with a measure of health behavior change. Random-effects meta-analysis was performed to examine whether the method of eHealth delivery impacted the level of engagement, behavior change, and health outcomes.

**Results:**

A total of 24 RCTs were included predominantly examining dietary and physical activity behavior change interventions. There were 11 studies that used a synchronous approach and 11 studies that used an asynchronous approach, whereas 2 studies used a combined delivery method. Use of eHealth interventions improved exercise behavior (standardized mean difference [SMD] 0.34, 95% CI 0.21-0.48), diet behavior (SMD 0.44, 95% CI 0.18-0.70), fatigue (SMD 0.21, 95% CI −0.08 to 0.50; SMD change 0.22, 95% CI 0.09-0.35), anxiety (SMD 1.21, 95% CI: 0.36-2.07; SMD change 0.15, 95% CI −0.09 to 0.40), depression (SMD 0.15, 95% CI 0.00-0.30), and quality of life (SMD 0.12, 95% CI −0.10 to 0.34; SMD change 0.14, 95% CI 0.04-0.24). The mode of delivery did not influence the amount of dietary and physical activity behavior change observed.

**Conclusions:**

Physical activity and dietary behavior change eHealth interventions delivered to patients with cancer or survivors have a small to moderate impact on behavior change and a small to very small benefit to quality of life, fatigue, depression, and anxiety. There is insufficient evidence to determine whether asynchronous or synchronous delivery modes yield superior results. Three-arm RCTs comparing delivery modes with a control with robust engagement reporting are required to determine the most successful delivery method for promoting behavior change and ultimately favorable health outcomes.

## Introduction

### Background

The World Health Organization describes electronic health (eHealth) as a cost-effective and secure way to use information and communication technologies for health [1]. eHealth broadly encompasses the provision of health care services, education, allows surveillance, and the development of knowledge and research through technology [1]. Mobile health (mHealth) is a subset of eHealth, where mobile devices support the delivery of medical and public health care to individuals and populations [2]. In 2007, the number of individuals using the internet around the world was approximately 1.3 billion [3]. Furthermore, in 2018, this number had trebled to 3.9 billion people with internet access (51.2% of the world’s population) [3,4]. The use of mHealth in the delivery of health interventions is increasing worldwide because of the rapid growth of internet use and leaps in technological advancements. Its potential to target previously hard-to-reach populations and the need for innovative approaches to deliver health promotion and interventions in the face of aging populations and health care budget constraints make it an attractive delivery method [2,5-8].

Behavior change interventions can be defined as “coordinated sets of activities designed to change specified behaviour patterns” [9]. The effectiveness of digital behavior change interventions to promote behavior change is likely to be dependent on a complex interplay of factors, which are still in their infancy in terms of understanding. A systematic review of behavior change interventions delivered via the internet found that more extensive use of underlying behavior change theory and utilization of more behavior change techniques were important factors in overall intervention effectiveness [10]. Although eHealth interventions offer the promise of enhancing health care to populations in rural and regional settings and overcoming some of the challenges associated with accessing traditional health care delivery modes, there has been concern that in some segments of the community, provision of eHealth may exacerbate already prominent inequalities [11]. Of particular concern are individuals who have low health literacy, access to technology, and familiarity and confidence in the use of technology [11-14].

eHealth interventions, focusing on behavior change, are being increasingly used in patients with cancer and survivors. In 2018, cancer rates around the world increased to 18.1 million cases per year with 1 in 6 women and 1 in 5 men receiving a diagnosis in their lifetime [15]. Cancer survivorship also increased, with 43.8 million people surviving up to 5 years [15]. Many of these individuals live with long-term treatment side effects, including cardiac dysfunction, functional decline often precipitated by chronic pain and fatigue syndromes, obesity, diabetes, osteoporosis, premature menopause, neurocognitive deficits, and risks associated with primary recurrence and second cancers [16-18]. There are well-established guidelines and recommendations to modify risks associated with physical activity and healthy eating behavioral patterns post cancer diagnosis [19-21]. Studies of behavior change interventions in this population often use a guideline as a basis to improve physical activity and healthy eating behaviors. A recent review of self-guided technology-supported nutrition and physical activity interventions in adults with cancer found benefits in physical activity and fatigue with some modest effect on dietary behaviors and health-related quality of life [22]. Another review looked at telephone, print, and web-based interventions and found that improvements were reported in 76% of the studies included for physical activity, dietary behaviors, or weight [23]. However, these reviews did not contrast the relative effectiveness of different approaches to delivering eHealth behavior change interventions. There are 3 important categories that eHealth interventions can be described: synchronous, asynchronous, and combined. Synchronous eHealth interventions are delivered via real-time interactions between the person and health care provider, encompassing face-to-face contact through teleconferencing equipment, telephone (telehealth), and live chat via web-based [24-26]. Asynchronous techniques include store-and-forward methods such as email and automated messaging systems without a live interaction component [24-26]. Combination approaches use both synchronous and asynchronous elements. These distinctions are important because they impact how health services need to structure and staff services that use these approaches and dictate whether or not health services have to rely on technology-based platforms to enable delivery.

### Objectives

There is a need to characterize effective eHealth interventions to enable improved design and implementation of evidence-based cancer health behavior change interventions, which will translate into the ability to scale up to affect health behavior change in a wide range of health promotion and health care management situations. This systematic literature review seeks to compare synchronous with asynchronous delivery modes and contrast their impact on behavior change and quality of life outcomes in adult patients with cancer or survivors. It also seeks to examine whether the degree of behavior change influences the amount of change in quality of life and to describe the behavior change theories and techniques used in the field.

## Methods

In conducting this review, we recognized that increased access to mobile internet technologies, and increased availability of health information on the web, combined with changing behaviors in accessing health information [27,28] means that people who previously may have relied on health professionals to provide them with information are now better enabled and more likely to find information on their own. We chose to restrict study selection to being from a contemporary period (from 2007 onward) under the justification that the substantial increase in availability of information about health behaviors and managing the lifestyle consequences of cancer through the internet has evidently changed how people may seek and find information about their health issues and respond to behavior change interventions delivered by distance.

### Reporting Guidelines

This systematic review follows the Cochrane Collaboration’s Handbook of Systematic Reviews of Interventions [29]. This systematic review was registered with The International Prospective Register of Systematic Reviews (PROSPERO) CRD42018103855. The methods used in this systematic review are in line with the preferred reporting items for systematic reviews and meta-analysis (PRISMA) guidelines. A completed copy of the PRISMA checklist is attached (Multimedia Appendix 1).

### Search Strategy

A three-part search strategy was used to identify studies that met the following inclusion criteria: (1) we searched electronic bibliographic databases for published work including SCOPUS, Ovid MEDLINE, Excerpta Medica dataBASE (EMBASE), Cumulative Index to Nursing and Allied Health Literature (CINAHL) Plus, PsycINFO, and Cochrane CENTRAL; and (2) we searched the reference lists of the primary studies included in the review. We undertook a second phase of study identification where we completed a hand search of the *Journal of Medical Internet Research Cancer* and expanded the database search to include PubMed with the additional search term “website” (Multimedia Appendix 2).

## Search Terms

### Example Search Strategy

Mobile app*, electronic mail, internet, mhealth, mobile health, ehealth, electronic health, telehealth, telemedicine, telenursing, telemonitoring, telerehabilitation, telephone, cell* phone*, cell* telephone*, mobile telephone, mobile phone*, smartphone*, email*, electronic messag*, electronic mail, text messag*, short messag* service*, SMS, MMS, interactive voice response, multimedia, web-based, automat* reminder*, videoconferenc*, online*

AND

behavio* chang*, health behavio* chang*, behavio* theory, behavio* modifi*, health promotion

AND

NOT child*, adolescen*, teen*, preschool*, infant*, toddler*

AND

specific validated database filters for randomised controlled trials

AND

English language, 2007 to current

The asterisk truncates the search term so that alternative terms are also identified (eg, behavio* will find behavior and behavioral).

Titles and abstracts were retrieved using the search strategy. Reference lists were then exported into Clarivate Analytics EndNote X8 and duplicates were removed. References were then exported into Covidence where further *deduplication* occurred. Abstracts were reviewed by the author (KF), and an independent author (MS) performed a parallel review. The overall review was limited to the population of people with cancer when the abstract screening resulted in over 400 studies to be reviewed. Papers where reviewers disagreed on the rating of eligibility criteria were re-examined and discussed to reach consensus. The full-text papers were then retrieved and independently assessed by 2 reviewers (KF and MS). Hand searching for primary studies included in the review resulted in a further 23 studies for review.

### Selection Criteria

Studies were limited to those relating to cancer patients or survivors, published with one or more search terms, were subjected to peer review, published in the English language, involved human adult subjects aged 18 years and over, and dated from 2007 to March 2019. The primary intervention was delivered through an eHealth delivery method such as telephone or internet, either asynchronous or synchronous or combined interventions against a control (including usual care or wait list control or no intervention), random assignment of participants to treatment or comparison groups, and a measure of health behavior change must have been taken after the intervention. Studies were excluded if there was any face-to-face component, as we sought to examine interventions purely delivered via eHealth approaches.

### Outcomes

The Kirkpatrick model [30] is an internationally recognized tool to evaluate the effectiveness of training interventions. In this review, training is defined as the health behavior change intervention. We sought to examine relationships between the intervention delivery mechanism (synchronous or asynchronous) and the 4 different levels of the Kirkpatrick model. The 4 levels of the model are as follows:

- Level 1 (reaction): This is how participants responded to the training. This review focuses on participant user engagement metrics to assess reaction.
- Level 2 (learning): This is the content learned from the training provided and is usually knowledge tests completed pre- and postintervention.
- Level 3 (behavior): This level examines the participants’ application of learning. We review the participants’ ability to translate the intervention into health-related behavior change.
- Level 4 (results): This is the degree to which targeted outcomes have occurred as a result of the training provided. This review looks at health outcomes as described by previous studies.

This review examined how the different delivery mechanisms of behavior change intervention not only impact the individual levels of this model but also impact the interaction between these levels. We did not extract data related to the second level of the Kirkpatrick hierarchy (learning) as behavior change interventions do not necessarily require new information to be learned to change behaviors (this is a minor limitation in applying an educational framework in a behavior change context).

### Primary Outcomes

The primary outcome for this review was defined as a change in health behaviors (eg, increases in moderate-to-vigorous physical activity or fruit and vegetable consumption) from baseline to the conclusion of the active intervention. This was chosen as it was most likely to be the time of greatest compliance with the intervention.

### Secondary Outcomes

The secondary outcomes for this review were engagement in the eHealth interventions and changes in quality of life, fatigue, anxiety, and depression. We sought to measure the proportion of participant initiation of the intervention, the frequency of intervention delivery per week, and duration of intervention delivery in minutes. Changes in quality of life, fatigue, anxiety, and depression were measured relative to the measure taken as close to the conclusion of the active intervention as possible, not after any period of follow-up.

### Other Descriptive Data

We sought to describe the behavior change theories used and behavior change techniques employed in eHealth interventions.

### Data Extraction

One (KF) and either of the 2 reviewers (MS or KH) independently extracted data including study identifiers, study design, population characteristics, consent and retention rate, intervention, behavior change and health outcomes of interest, behavior change theory and techniques used, intervention participation rates, control condition details, length of follow-up, and information to complete a risk of bias assessment into a standardized data extraction table. Data were extracted on the key outcomes (objective or self-reported) defined by the study, including those that reported on multiple behavior change outcomes (eg, diet and physical activity). Where this was not clear, 2 reviewers independently chose the most relevant behavior change outcome to the study. Where data were incompatible for meta-analysis, authors were contacted twice via email, and 2 out of 9 authors who were contacted responded with forthcoming information. Engagement data were also extracted by 2 reviewers.

#### Risk of Bias Assessment

Two review authors (KF and KH) independently assessed the risk of bias for randomized controlled trials (RCTs) using the Cochrane Collaboration’s tool for assessing bias [31]. Differences of opinion were discussed and agreed upon between the 2 reviewers.

### Analysis

#### Behavior Change and Health Outcomes

Data were separated into groups of similar outcomes to perform pooled random effects meta-analysis using standardized mean difference (SMD) of summative level data. Standardized effect sizes were considered small at 0.2, moderate at 0.5, and large at 0.8 [32]. A random effects metaregression analysis was undertaken to determine if the mode of delivery influenced the amount of behavior change observed. The SD of the effect size was imputed for one study [33], as variance data were not available. The imputation method was used to borrow a particular value from other studies using the same outcome measure [34]. The functional assessment of chronic illness therapy-fatigue, used in 3 studies [35-37], was transformed by multiplying the mean values by −1 to ensure that all scales were in the same direction [29]. For visual representation and ease of interpretation, fatigue, depression, and anxiety were transformed to allow lower scores to represent improved outcomes.

#### Initiation of Engagement Data

Variances of raw proportion data were transformed using the Freeman-Tukey arcsine square root transformation [38]. The DerSimonian-Laird random effects model [39] was then used to pool these transformed proportions to provide a measure of the extent of between-study heterogeneity. The Wilson score was then used to provide CIs for the pooled estimate [40]. A random effects plot was then created. Random effects metaregression analyses were then performed to determine whether any of the different delivery modes influenced the amount of engagement initiation.

## Results

### Summary

**Figure 1:**
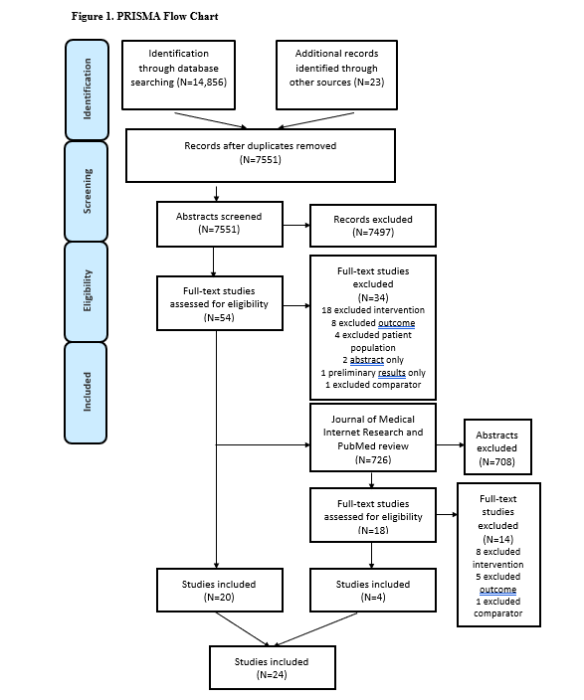
Preferred reporting items for systematic reviews and meta-analysis flowchart.

The literature search identified a total of 15,582 studies with a further 23 studies identified through reference list searching of relevant papers. After EndNote X8 and Covidence deduplication, a total of 8259 studies were screened, with 72 s remaining for full-text screening. A total of 24 studies were included in this review; 11 studies were delivered via synchronous and asynchronous methods, respectively, with 2 studies using a combined approach (Figure 1). The studies were predominantly delivered in the United States with 13 studies [37,41-52]; 3 in Australia [35,36,53], South Korea [33,54,55], and the Netherlands [56-58]; and 1 in Canada [59] and France [60]. The total sample size was 4583, ranging from n=18 to n=641. There were a range of different cancer types targeted during the study interventions: 7 unspecified cancer types [45,49,50,52,55-57,60]; 6 breast cancer [33,35,42,51,54,61]; 2 colon or colorectal [36,43]; 2 breast, prostate, or colorectal [44,59]; 2 melanoma [41,53]; and 1 each of breast/colon or rectal [37], colorectal or prostate [58], prostate [46], and urothelial cell carcinoma [47]. The majority of studies focused on posttreatment (also reported as survivors) with 17 studies [33,36,37,41,43-45,49-56,59,61], 6 studies included patients during active cancer treatment (2 used survivors and active cancer treatment) [35,42,47,57,58,60], and 1 study included patients undergoing surveillance for their cancer [46]. Of the 24 studies included in this review, 19 provided data on a measure of physical activity behavior change [33,35-37,42-45,48-50,54-57,59] and 9 studies provided data on a measure of dietary behavior change [33,36,42,44-47,54,56]; 2 studies provided data on skin self-examination during melanoma-related interventions [41,53]; 2 studies provided data on smoking cessation [52,56], whereas another study provided information on alcohol intake [36]. Interventions delivered via synchronous modes included telephone, Skype, and videoconferencing, whereas those delivered via asynchronous modes used combinations of custom or existing websites and mobile apps, with short messaging and email. Studies that used combined methods used web-based intervention and an online moderated forum, and telephone and SMS text messaging. Many studies in any of the delivery method intervention groups used adjunct features, including pedometers and written workbooks (Multimedia Appendix 3).

### Behavior Change Techniques

The use of behavior change techniques varied significantly between studies; studies used between 2 and 17 (median 8) different techniques. The most popular behavior change techniques used across the studies were goal setting (behavior; 87%) and self-monitoring of behavior (85%), information about health consequences (70%), problem solving (67%), action planning (62%), feedback on behavior (61%), and instructions on how to perform behavior (61%). Out of the 93 hierarchically set behavior change techniques from the Michie et al [62] taxonomy, only 33 were used across the 24 studies included in this review (Multimedia Appendix 4).

### Behavior Change Theory

A total of 20 studies reported on the use of behavior change theory. Within the studies that reported on behavior change theory, social cognitive theory and the transtheoretical model of change were the 2 most popular theories used, with 60% and 39% of papers reporting on these, respectively. A total of 8 studies (33%) used more than one theory to underpin their interventions (Multimedia Appendix 4).

### Risk of Bias Assessment

**Figure 2:**
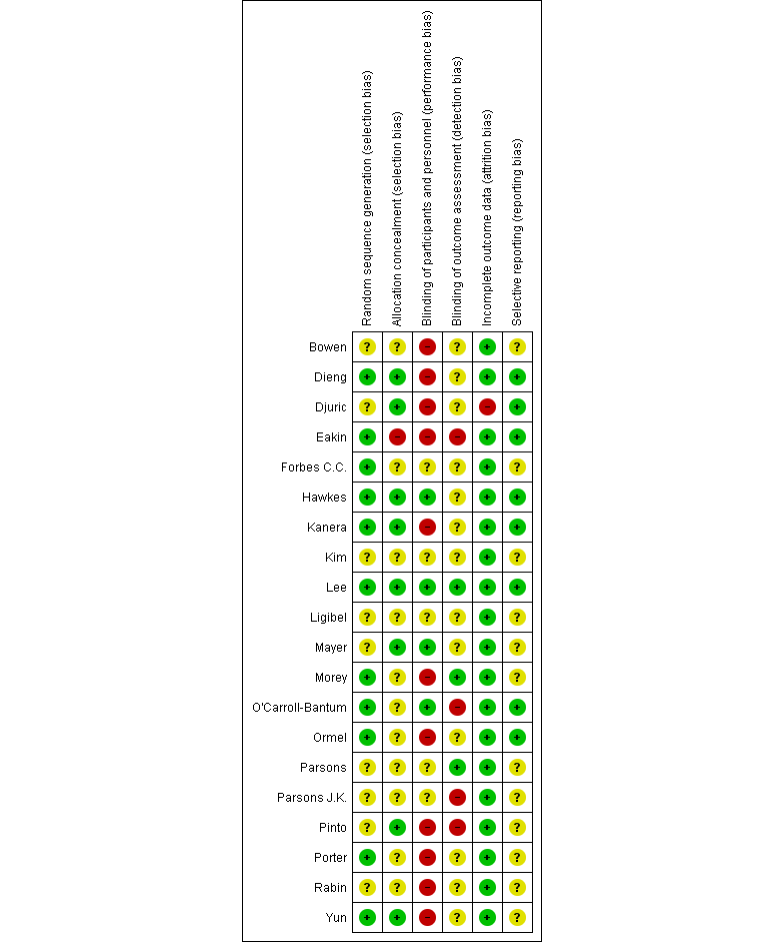
Risk of bias summary of the included randomized control trials using the Cochrane Collaboration risk of bias tool.

**Figure 3:**
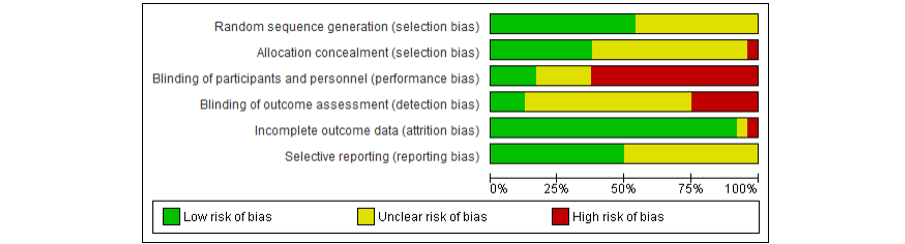
Risk of bias graph.

The outcome of the risk of bias assessment is presented in Figures 2 and 3. Attrition bias was considered to be low across most studies. There was a high degree of risk associated with blinding of personnel, participants, and outcome assessment. The reporting of study-related processes was highly variable, which led to many areas of the risk of bias being assigned as unclear.

### Behavior Change: Physical Activity

**Figure 4:**
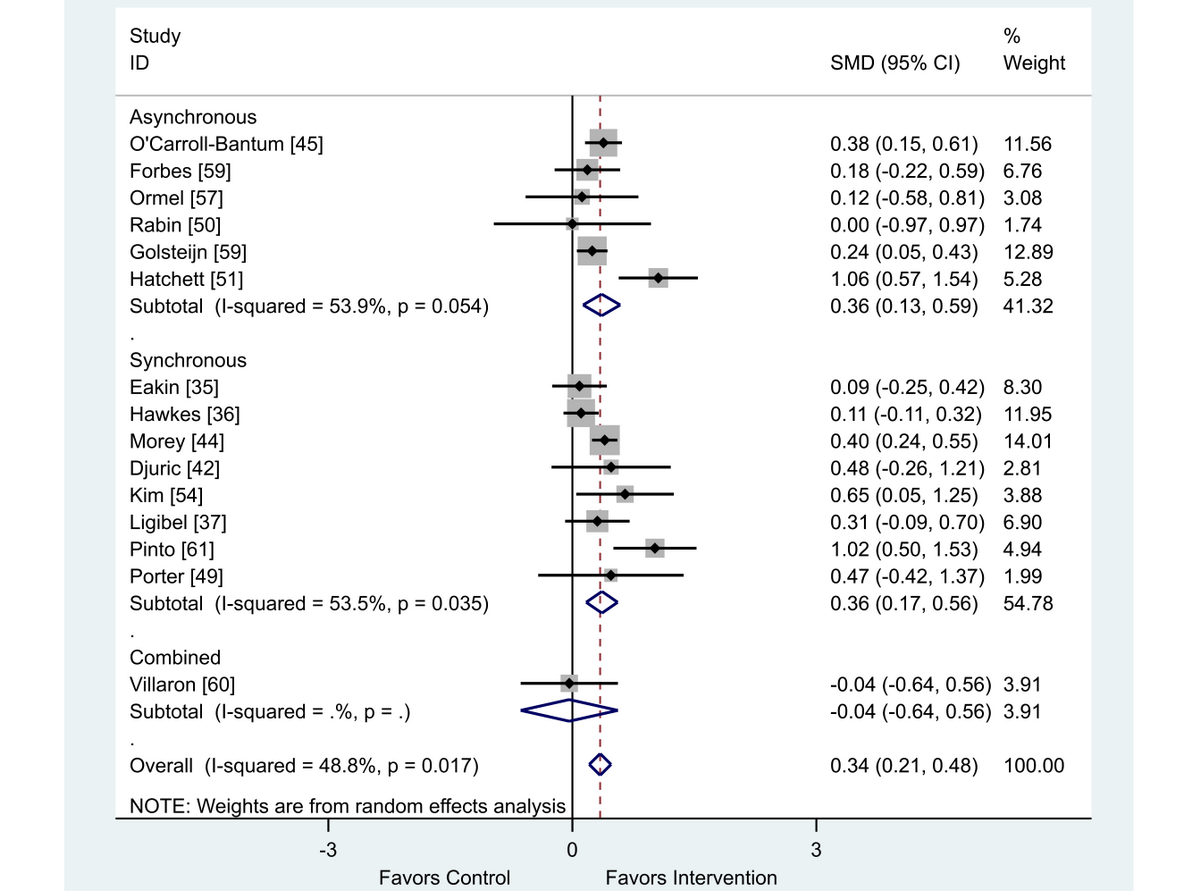
Effect of electronic health interventions compared with a control on physical activity interventions, analyzed using standardized mean difference. SMD: standardized mean difference.

Of the 19 studies in this review that presented data on physical activity as a behavior change outcome, 15 studies were included in this meta-analysis [35-37,42,44,45,48-51,54,57-60] and a further 4 could not be included as the data were presented in a format incompatible with meta-analysis [33,43,55,56]. There was a finding in favor of eHealth interventions (SMD 0.34; 95% CI 0.21 to 0.48) for increasing physical activity behaviors (Figure 4).

When analyzed by delivery method, the intervention delivery mode of synchronous, asynchronous, or combined did not impact the overall positive outcome effect (Table 1).

**Table 1:** Metaregression comparing the effect of synchronous versus asynchronous versus combined electronic health interventions on physical activity behavior change, analyzed using standardized effect size.

Standardized effect size	Coefficient	95% CI	*P* value
Asynchronous	−0.01	−0.38 to 0.35	.95
Combined	−0.41	−1.27 to 0.45	.32
Constant	0.37	0.13-0.61	.005

The 4 studies examining asynchronous interventions provided data that were presented in a format incompatible with meta-analysis. One reported a significant increase in moderate physical activity in their intervention group (*P=*.04); however, the authors reported that this did not remain significant after controlling for multiple testing [56]. Another study reported that the intervention increased the proportion of participants who undertook moderate intensity aerobic exercise for at least 150 min per week to a greater extent than the control (*P=*.001) [33]. These outcomes contrasted with 2 other studies that reported no effect in improving the secondary outcome of metabolic equivalent of task (*P=*.48) [55] and the proportion of participants who undertook moderate-to-vigorous physical activity (*P=*.12) [43].

### Behavior Change: Dietary

**Figure 5:**
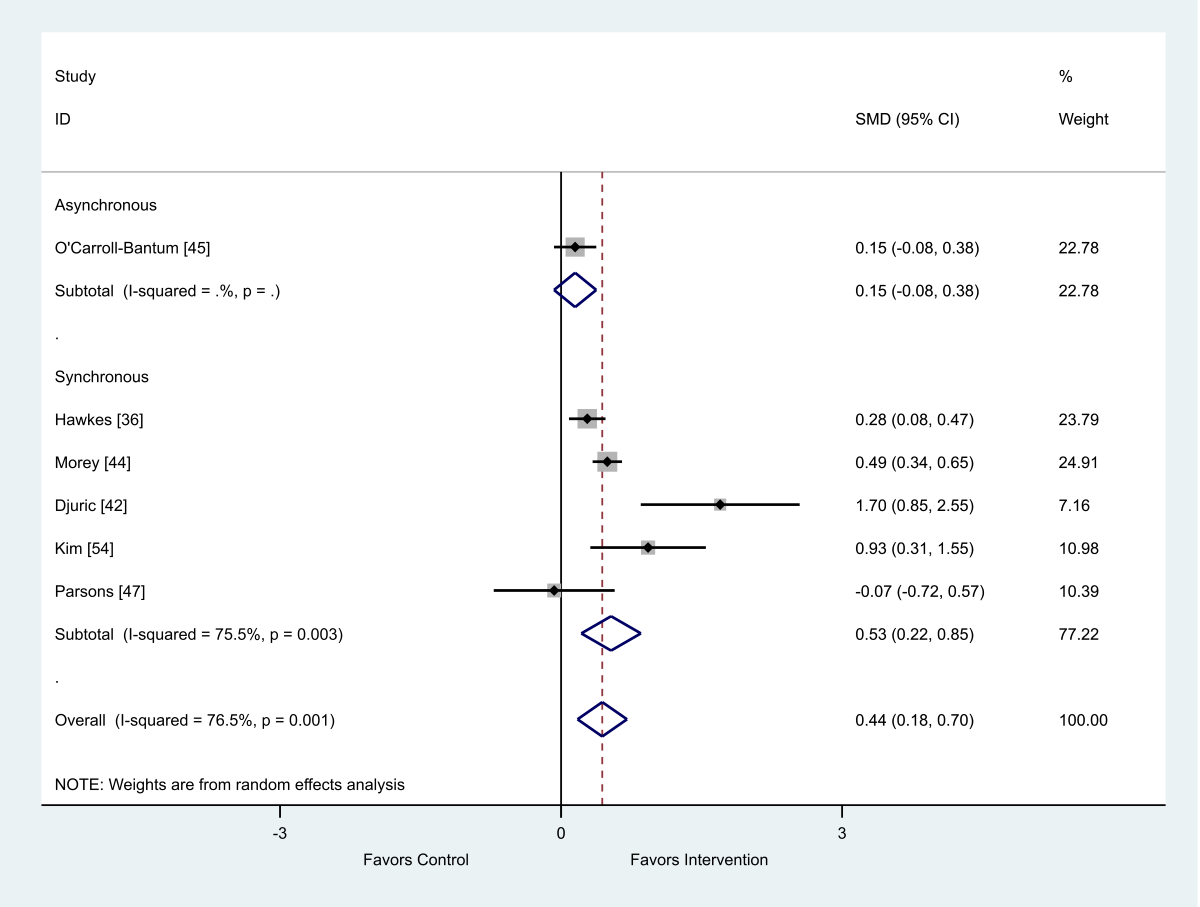
Effect of electronic health interventions compared with control on dietary interventions, analyzed using standardized mean difference. SMD: standardized mean difference.

A total of 9 studies included in this review provided data on diet as a behavior change outcome; 6 studies were included in this meta-analysis [36,42,44,45,47,54] and a further 3 could not be included as the data were presented in a format incompatible with meta-analysis [33,46,56]. There was a finding that diet interventions delivered via eHealth can improve behavior change compared with control (SMD 0.44; 95% CI 0.18 to 0.70; Figure 5).

When analyzed by delivery method, the intervention delivery mode of synchronous or asynchronous did not impact the overall positive outcome effect (Table 2).

**Table 2:** Metaregression of synchronous and asynchronous dietary interventions on behavior change outcome, analyzed using standardized effect size.

Standardized effect size	Coefficient	95% CI	*P* value
Asynchronous	−0.44	−2.18 to 1.29	.52
Constant	0.60	−0.17 to 1.36	.10

In total, 3 studies provided data incompatible with the meta-analysis. The 2 studies using asynchronous delivery approaches reported significantly higher vegetable consumption in participants accessing a web-based guide (*P=*.02), which the authors reported did not remain significant after accounting for multiple testing [56], whereas dietary quality index was greater in the intervention compared with the control in the other study (*P=*.001) [33]. One synchronous intervention reported a significant increase in alpha-carotenoid concentrations compared with controls (*P*<.05) [47].

### Other Behavior Change Outcomes

A total of 5 studies provided data on other primary behavior change outcomes, including smoking cessation, alcohol intake, and skin self-assessment. A study examining asynchronous interventions reported no effect on smoking cessation (*P=*.28; odds ratio [OR] 2.61) [56]. Similarly, no improvement in smoking cessation rates was found in a study using a combined delivery mode approach [52]. A separate trial using an asynchronous delivery mode to encourage skin self-examination was effective (OR derived from percentage data provided 1.90, 95% CI 2.23-2.94) [41]. Conversely, another study examining the effect of an intervention (delivered synchronously) to encourage skin self-examination reported a decrease in the reported rate of skin self-examination in the intervention group compared with control (adjusted between-group difference −0.13; 95% CI −0.4 to 0.2; *P=*.40); however, this outcome was measured at 6 months follow-up rather than immediately post intervention at 1 month. This study also presented information about melanoma-related knowledge change and was the only study included in this review that presented any data on knowledge change. They found an improvement in melanoma-related knowledge in the intervention group at 6 months using an adjusted between-group difference (1.7; 95% CI 0.8-2.6; *P*<.001) [53]. The final study examining a synchronously delivered intervention revealed no significant difference between groups on alcohol intake (grams per day; *P=*.26) [36].

### Health Outcome: Quality of Life

**Figure 6:**
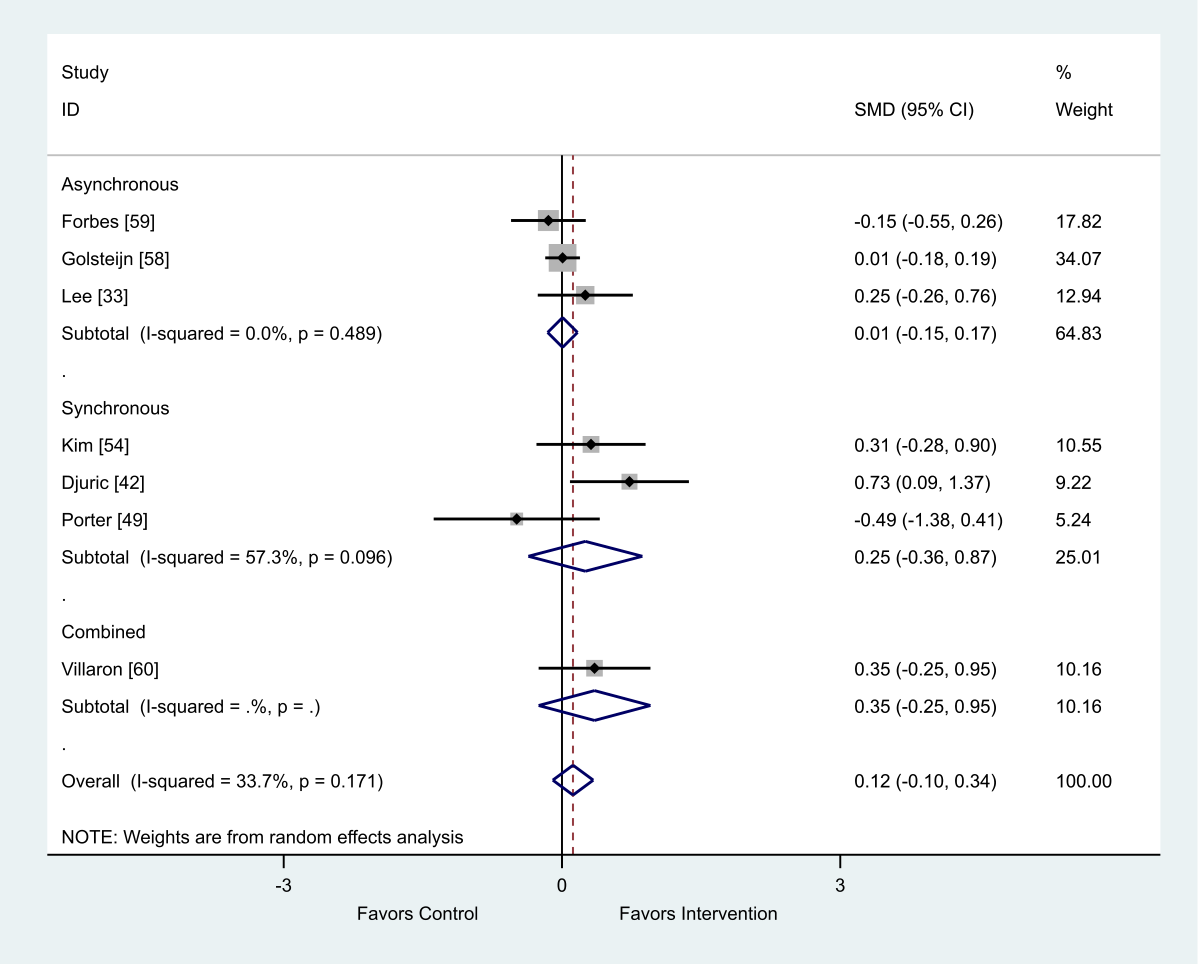
Effect of electronic health interventions compared with a control on quality of life interventions, analyzed using standardized mean difference (postintervention mean and SD). SMD: standardized mean difference.

There were 13 studies that provided data on quality of life [33,35-37,42-44,49,54,55,58-60]; 2 meta-analyses were performed as data were presented as a combination of final scores and change scores. Of the 7 studies that provided final scores, there was a favorable impact of the intervention for the synchronous (SMD 0.25; 95% CI −0.36 to 0.87) and combined (SMD 0.35; 95% CI −0.25 to 0.95) eHealth interventions on quality of life. The 3 studies using an asynchronous mode for intervention delivery reported no improvement in quality of life compared with control conditions (SMD 0.01; 95% CI −0.15 to 0.17; Figure 6).

**Figure 7:**
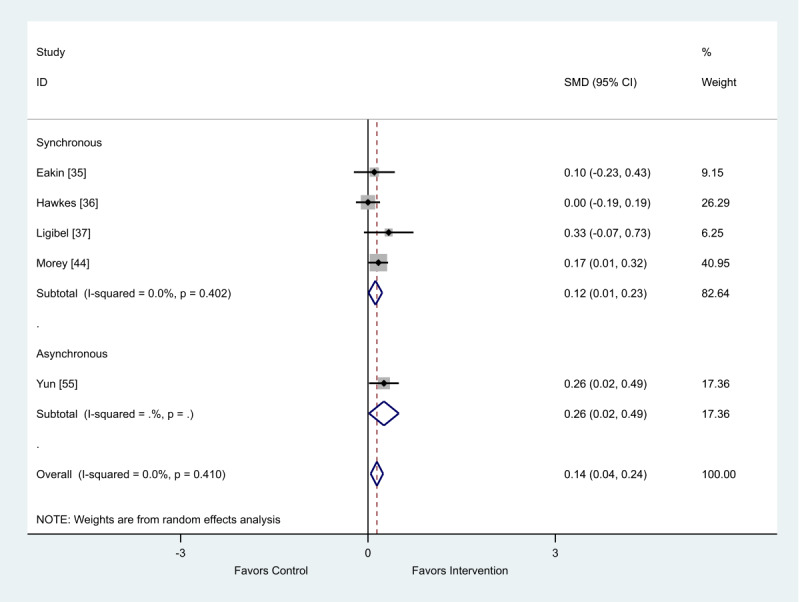
Effect of electronic health interventions with a control on quality of life interventions, analyzed using standardized mean difference (postintervention mean and SD change scores). SMD: standardized mean difference.

Of the 5 studies that provided change scores, there was a favorable impact of the intervention for both asynchronous (SMD 0.12; 95% CI 0.01-0.23) and synchronous (SMD 0.26; 95% CI 0.02-0.49) eHealth interventions on quality of life (Figure 7).

One study provided data that were incompatible with the meta-analysis. It was found that quality of life was not significantly different between the asynchronous intervention group and the no intervention control group over the intervention period. The intervention group baseline outcome score was 105 versus the postintervention outcome score of 109.1 and the control group baseline outcome score of 103.3 versus the postintervention outcome score of 106.5 [43].

When analyzed by delivery method, the intervention delivery mode of synchronous or asynchronous did not impact the overall positive outcome effect (Table 3).

When analyzed by delivery method, the intervention delivery mode of synchronous or asynchronous did not impact the overall positive outcome effect (Table 4).

**Table 3:** Metaregression of synchronous and asynchronous interventions on quality of life outcome, analyzed using standardized effect size.

Standardized effect size	Coefficient	95% CI	*P* value
Asynchronous	−0.31	−1.05 to 0.43	.31
Combined	0.04	−1.22 to 1.29	.94
Constant	0.31	−0.37 to 1.0	.27

**Table 4:** Metaregression of synchronous and asynchronous interventions on quality of life outcome, analyzed using standardized effect size (postintervention mean and SD change scores).

Standardized effect size	Coefficient	95% CI	*P* value
Asynchronous	0.14	−0.31 to 0.58	.40
Constant	0.12	−0.70 to 0.31	.14

### Health Outcome: Fatigue

**Figure 8:**
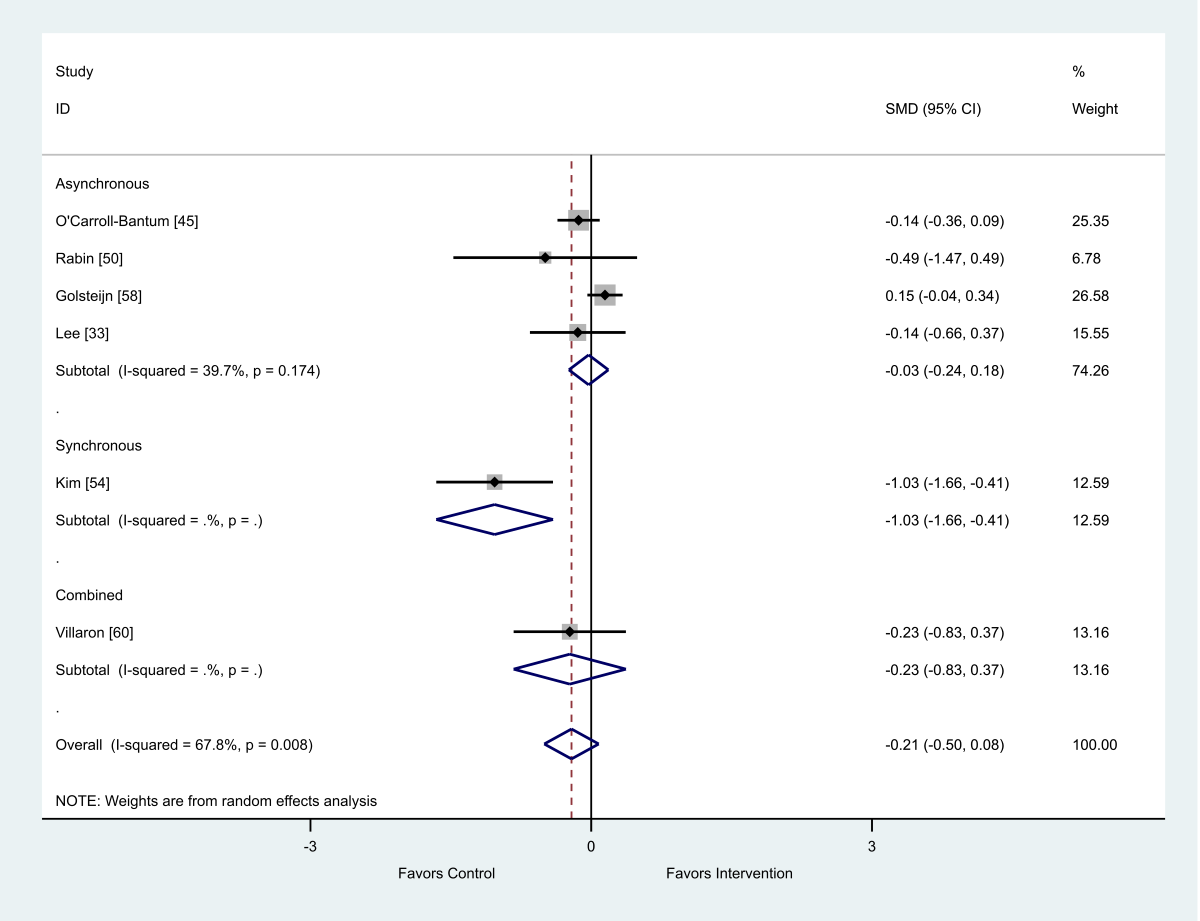
Effect of electronic health interventions with a control on fatigue interventions, analyzed using standardized mean difference (postintervention mean and SD). Lower scores indicate improved fatigue. SMD: standardized mean difference.

There were a total of 10 studies that presented data on fatigue [33,35-37,45,50,54,55,58,60,63]. Of the 6 studies using final scores, synchronous (SMD 1.03; 95% CI 0.41-1.66) and combined (SMD 0.23; 95% CI −0.37 to 0.83) interventions showed a favorable impact on fatigue. The asynchronous group reported no intervention effect on fatigue (SMD 0.03; 95% CI −0.18 to 0.24; Figure 8).

**Figure 9:**
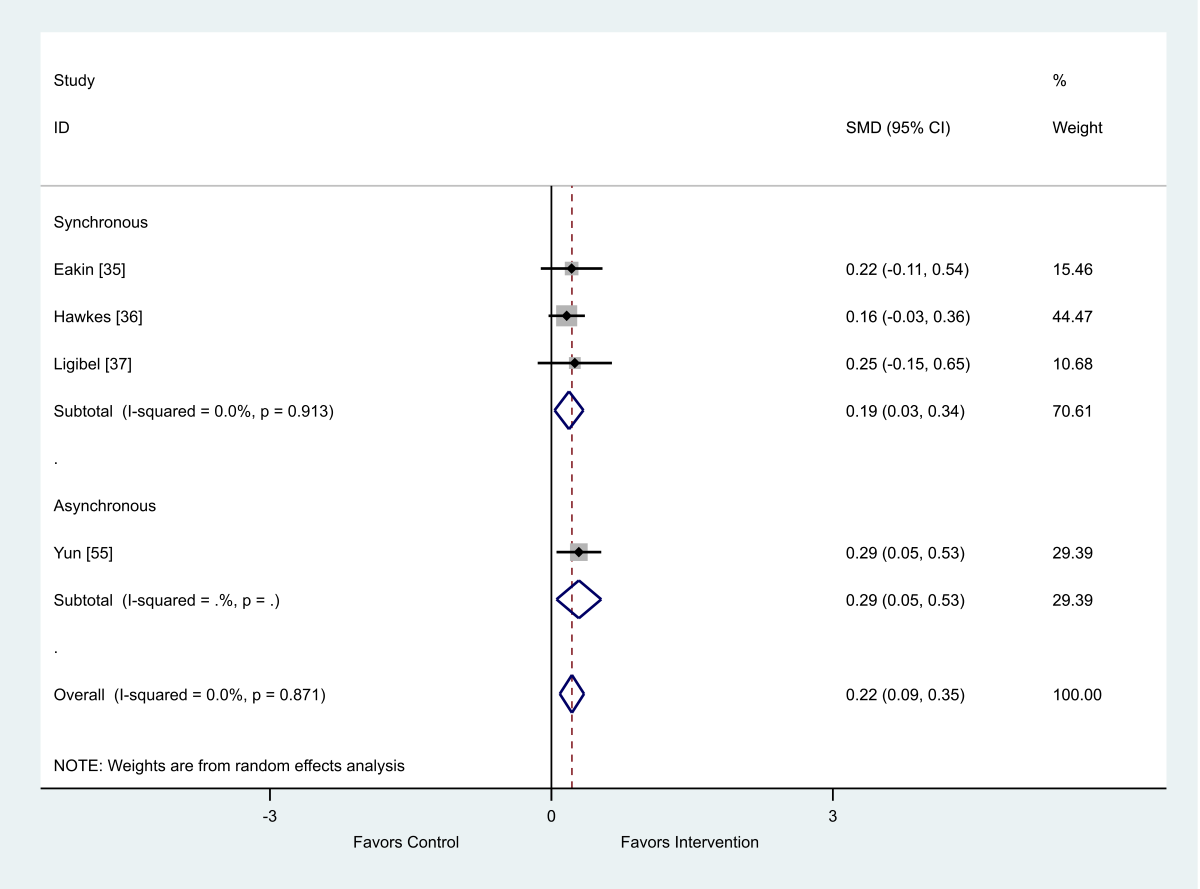
Effect of electronic health interventions with a control on fatigue interventions, analyzed using standardized mean difference (postintervention mean and SD change scores). Lower scores indicate improved fatigue. SMD: standardized mean difference.

Of the 4 studies that provided change scores, both synchronous (SMD 0.19; 95% CI 0.03-0.34) and asynchronous (SMD 0.29; 95% CI 0.05-0.53) eHealth interventions showed a favorable impact on fatigue (Figure 9).

When analyzed by delivery method, the intervention delivery mode of synchronous, asynchronous, or combined did not impact the overall positive outcome effect (Table 5).

When analyzed by delivery method, the intervention delivery mode of synchronous or asynchronous did not impact the overall positive outcome effect (Table 6).

**Table 5:** Metaregression of synchronous and asynchronous interventions on fatigue outcome, analyzed using standardized effect size.

Standardized effect size	Coefficient	95% CI	*P* value
Asynchronous	1.00	−0.16 to 2.17	.07
Combined	0.80	−0.74 to 2.35	.20
Constant	−1.03	−2.14 to 0.77	.08

**Table 6:** Metaregression of synchronous and asynchronous interventions on fatigue outcome, analyzed using standardized effect size.

Standardized effect size	Coefficient	95% CI	*P* value
Asynchronous	0.11	−0.51 to 0.73	.54
Constant	0.19	−0.15 to 0.53	.14

### Health Outcome: Depression

**Figure 10:**
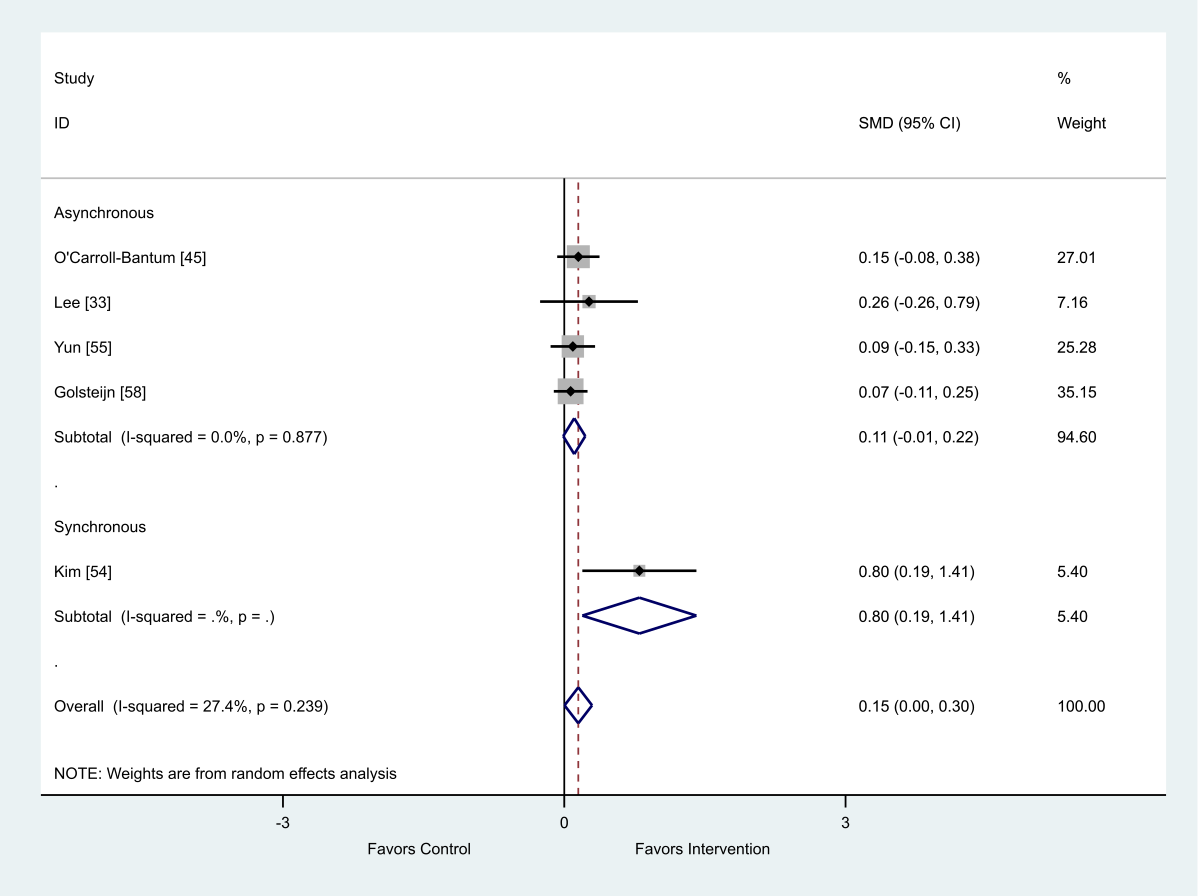
Effect of electronic health interventions with a control on depression interventions, analyzed using standardized mean difference (postintervention mean and SD). Lower scores indicate improved depression. SMD: standardized mean difference.

There were a total of 6 studies that presented data on health outcome depression [33,45,53-55,58]. Of the 5 studies’ final scores, the synchronous intervention showed a favorable impact on depression (SMD 0.80; 95% CI 0.19-1.41). The asynchronous group reported no intervention effect on depression (SMD 0.11; 95% CI −0.01 to 0.22; Figure 10).

The one that compared asynchronous study with a no intervention control that reported change scores found no intervention effect on depression (group difference −1.0; 95% CI −2.8 to 0.8; *P=*.40).

When analyzed by delivery method, the intervention delivery mode of synchronous or asynchronous did not impact the overall positive outcome effect (Table 7).

**Table 7:** Metaregression of synchronous and asynchronous interventions on depression outcome, analyzed using standardized effect size.

Standardized effect size	Coefficient	95% CI	*P* value
Asynchronous	−0.69	−1.70 to 0.32	.12
Constant	0.80	−0.19 to 1.79	.08

### Health Outcome: Anxiety

There were 6 studies that provided health outcome data on anxiety [33,35,53-55,58].

**Figure 11:**
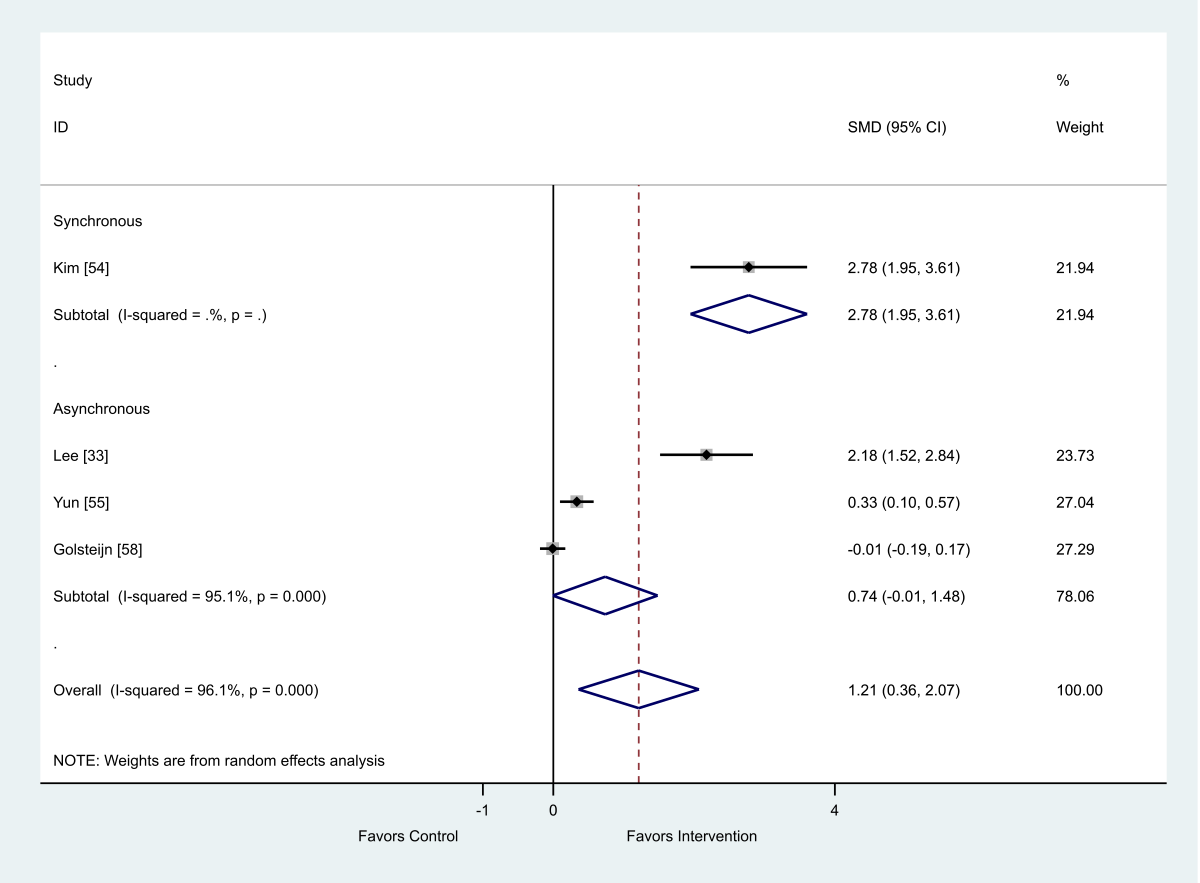
Effect of electronic health interventions versus control group on anxiety interventions, analyzed using standardized mean difference (postintervention mean and SD). Lower scores indicate improved anxiety. SMD: standardized mean difference.

Of the 4 studies that provided final scores, both synchronous (SMD 2.78; 95% CI 1.95-3.61) and asynchronous (SMD 0.74; 95% CI −0.01 to 1.48) modes showed a favorable impact on anxiety (Figure 11).

**Figure 12:**
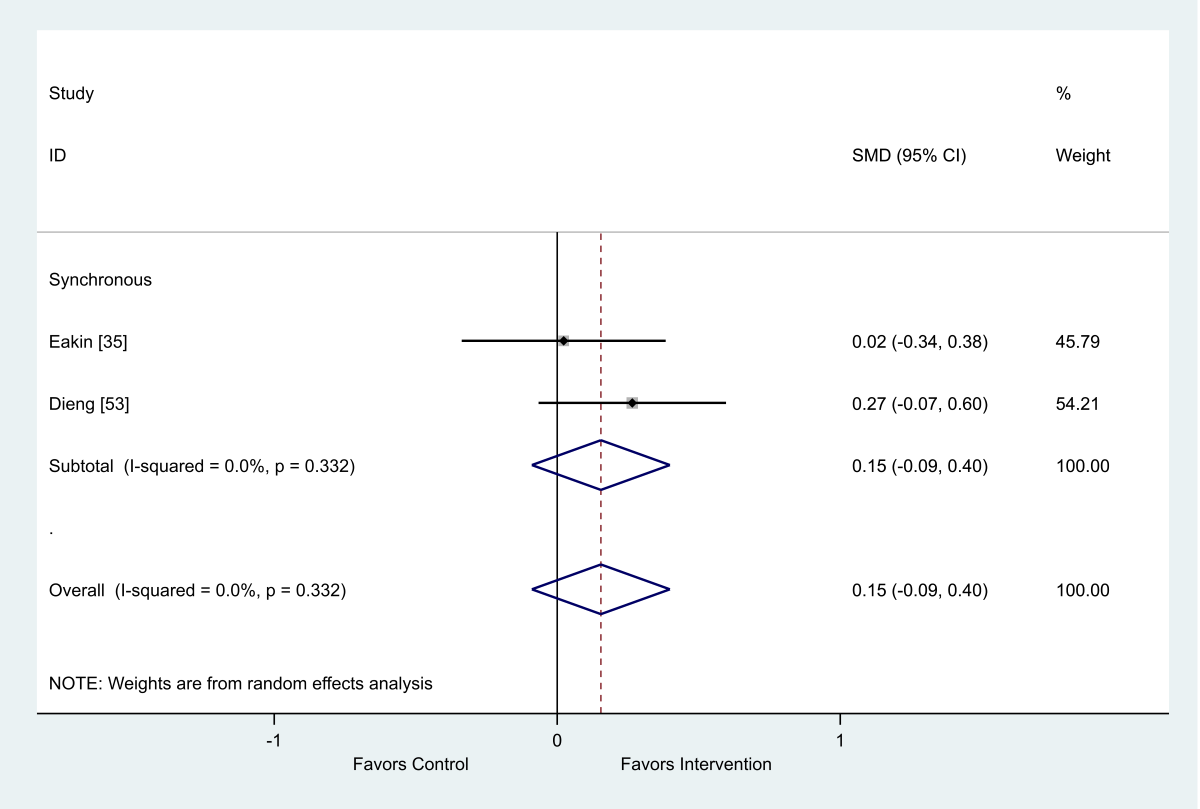
Effect of electronic health interventions with a control on anxiety interventions, analyzed using standardized mean difference (postintervention mean and SD change scores). Lower scores indicate improved anxiety. SMD: standardized mean difference.

Of the 2 studies that provided change scores comparing synchronous interventions with usual care control groups, there was a very small favorable intervention effect on anxiety (SMD 0.15; 95% CI −0.09 to 0.40; Figure 12).

When analyzed by delivery method, the intervention delivery mode of synchronous or asynchronous did not impact the overall positive outcome effect (Table 8).

**Table 8:** Metaregression of synchronous and asynchronous interventions on anxiety outcome, analyzed using standardized effect size.

Standardized effect size	Coefficient	95% CI	*P* value
Asynchronous	−1.98	−7.96 to 4.01	.29
Constant	2.77	−2.47 to 8.02	.15

We were unable to perform metaregression to examine the association between mode of delivery and change in anxiety, as there was only 1 delivery mode (synchronous) included in this meta-analysis.

### Initiation of Engagement Data

**Figure 13:**
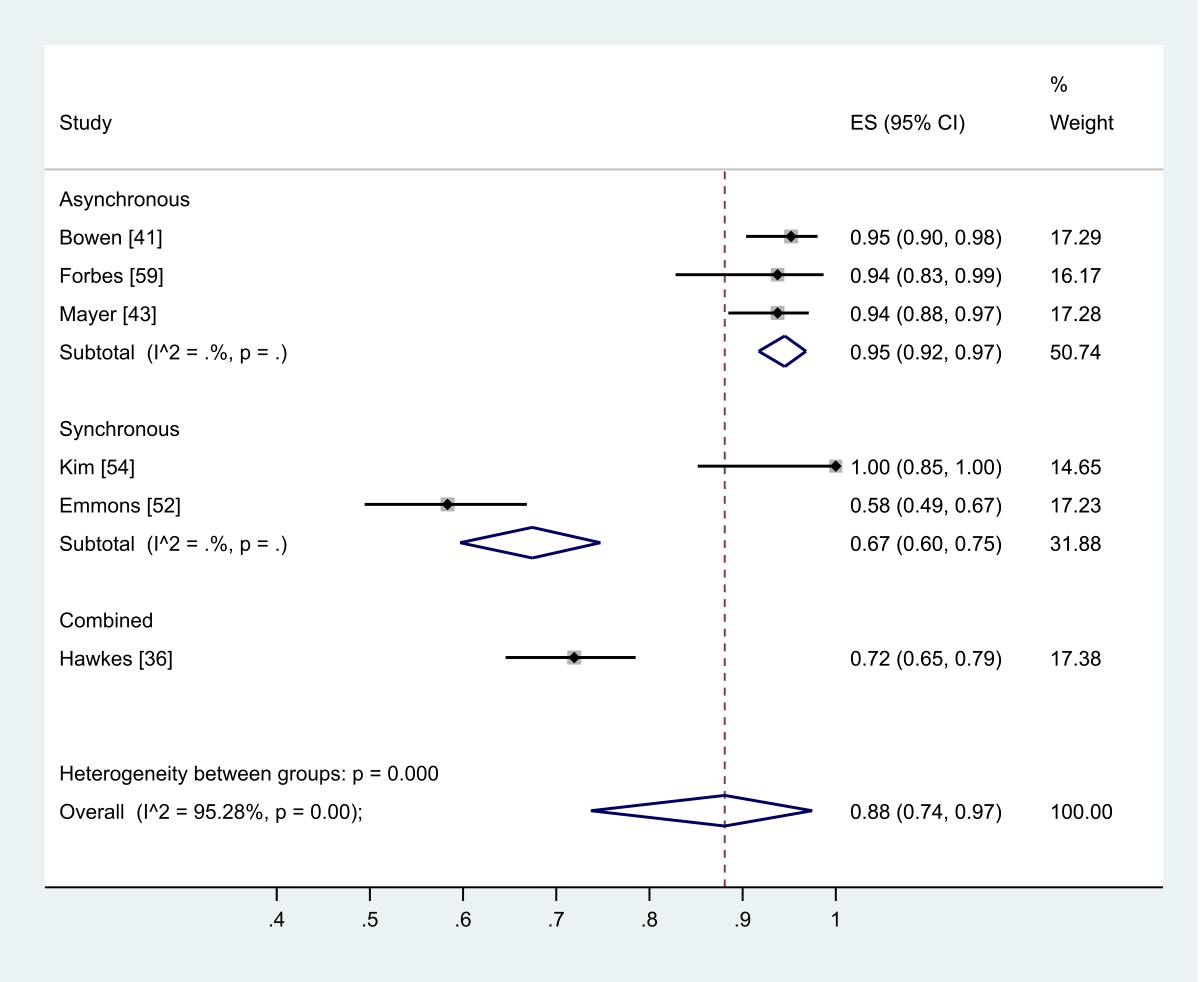
The effect of mode of delivery (synchronous or asynchronous) interventions on initiation engagement, analyzed using proportion.

A total of 5 studies did not provide any information about engagement or participation in their intervention [46,48,51,57,58]. Only 6 studies provided data on the initiation of participants to their interventions [36,41,43,52,54,59]. Other data pertaining to participant engagement or participation in interventions are shown in Table 1. The pooled effect of the proportion of initiation engagement found that 88% of participants commenced the intervention (Figure 13). Of the 11 synchronously delivered interventions, 9 studies reported on completion of telephone or videoconference sessions. In all, 7 studies reported that participants completed between 62% and 100% of all planned intervention sessions [33,36,42,45,49,53]. One study reported that 79% completed >75% of telephone calls [35] and another reported a median 9 out of 10 to 11 planned telephone calls completed [37]. Comparison of engagement and participation was made particularly challenging in the asynchronous group due to the heterogeneous nature of the reporting. Studies presented a range of data that included initiation, content accessed, log-in averages, and intervention fidelity or adherence.

## Discussion

### Principal Findings

The use of eHealth interventions improved physical activity, diet behaviors, quality of life, fatigue, depression, and anxiety in studies conducted across people who had been treated for a range of cancer types compared with control conditions. The overall impact appeared to be greater for the behavior change outcomes than the health outcomes. It did not appear that the mode of delivery (synchronous vs asynchronous vs combined) affected how much improvement the eHealth interventions generated. There was insufficient evidence to identify that the amount of behavior change was associated with the amount of change in health outcomes, although this may yet be identified as further studies report on both outcomes. These findings indicate that eHealth interventions may be beneficial for improving health behaviors and health outcomes when provided to cancer patients and/or survivors. However, there was one behavior change area where a negative impact on a health behavior (skin self-examination for melanoma) was reported, indicating that not all health behaviors may be improved from exposure to eHealth interventions.

At this stage, there is insufficient evidence to determine what delivery methods work for who, in what context, at what time. Therefore, the decision as to whether health services should provide synchronous or asynchronous eHealth behavior change interventions cannot yet be answered. None of the studies in this review compared synchronous with asynchronous eHealth approaches in the same population, using the same behavior change theory and techniques. A study that examined a telephone-delivered versus an internet-delivered weight loss intervention in cancer survivors found that the telephone group not only had greater losses in waist circumference (−0.75 vs −0.09; *P=*.03) but also had higher levels of engagement (80% completed calls vs 27% web log-ins) [64]. Other considerations, such as the cost-effectiveness and reliability of the different delivery approaches, should also come into play when making this decision. These issues were beyond the scope of this review but should be examined in future work to help inform this decision making.

We identified that initial engagement was high across the few studies that reported this outcome. We would also have liked to examine long-term engagement and the effect of this on intervention success, but the highly inconsistent and sporadic reporting of long-term engagement within included studies made synthesizing this information impossible in this review. There is no consensus regarding the definition of engagement in the context of eHealth and how to measure it, as many studies of behavior change interventions do not report at all on their participant engagement characteristics, yet report on attrition [65-69]. People could report absolute frequencies of contact, and where relevant, duration of contact. However, a recent new concept of effective engagement outlines that for each different intervention delivered, and for each individual person receiving the intervention, their requirement to engage and over what period of time will be different to achieve the intended outcome [70]. This concept indicates that *absolute* measures of engagement in eHealth interventions may not tell the full story as to whether the individual has engaged to a degree that is optimal for them. Perski et al [71] proposed that engagement with digital behavior change interventions occurs through specific direct and indirect mechanisms of action. Constructs of content, delivery, context, target behavior, population, and setting were proposed as important mechanisms to understand and report on when understanding engagement.

Our findings contrast with a previous review that looked at telephone interventions on physical activity and dietary behavior change in the population without cancer [72], in that the magnitude of effect appears to be lower than previously pooled analysis of behavior change outcomes using telephone interventions (effect size 0.60; 95% CI 0.24 to 1.19). It is plausible that the benefit of eHealth interventions in cancer populations may be diminished compared with populations without cancer because of concurrent treatment side effects and the impact that a cancer diagnosis can have on the capacity of patients to absorb new information [73]. A systematic review [74] of the effectiveness of mHealth technology use in behavior change interventions demonstrated mixed results. Studies reporting benefits described small effect sizes that were retained only in the short term. An older review [10] of interventions promoting health behavior change via the internet also examined the mode of delivery on efficacy and included 3 categories: automated functions, communicative functions, and supplementary modes. This review found that interventions were more effective in eliciting behavior change when there was more extensive use of underlying behavior change theory, more techniques of behavior change used in the delivery of the intervention, and additional methods of interacting with participants (combined techniques). This review found that between 2 and 17 behavior change techniques were used in each study, and a range of theoretical underpinnings was employed. This variability may also have contributed to the variability seen in the results of this review. These previous reviews were limited by not examining the potential mechanisms of action leading to the behavior change or the moderating effect of how the interventions were delivered.

We acknowledge a number of limitations on how we decided to undertake this review. Many of the studies included in this review used self-reported outcome measures with many failing to also include objective measures to corroborate data. We also combined both objective and subjective measures of behavior in the same meta-analysis. The small number of studies and high heterogeneity of data precluded a multiple metaregression to assess the interaction between behavior change and intervention delivery mode on health outcomes. Response bias, including social desirability bias, occurs frequently where self-reported outcome measures are used in research [75]. Ecological fallacy could also explain why we did not find a relationship between the amount of behavior change and the amount of change in quality of life. This could have been brought about by the examination of study level comparisons of analytic approaches and relationships rather than at the person level. We need to be able to trace the health behavior change data to the individual participants to understand the impact of the intervention.

Many of the studies reported multiple outcome measures, each with differing results. In this review, we chose to use data from the primary outcome measures (where reported) as the outcome of choice for behavior change and health outcomes. Where this was not reported, we decided to choose the outcome that we believed to be the most relevant outcome to the intervention examined in that particular study. We chose not to conduct metaregression analyses comparing synchronous and asynchronous eHealth approaches for outcomes where only one study was available in either of these subgroups. Such analyses would arguably have had limited generalizability and were at high risk of committing a type II statistical error. We chose to include studies within this synthesis regardless of the type of cancer involved or of the behavior change theories and techniques employed. Each theory and technique may have a different effect on the outcomes we examined and may be a source of confounding for our comparisons of synchronous and asynchronous eHealth approaches. Similarly, the type of patient population may have a moderating effect on the efficacy of synchronous and asynchronous eHealth interventions. Ideally, a comparison of synchronous and asynchronous eHealth approaches would be undertaken in the same populations, using the same behavior change theories and techniques. However, no such studies were identified in this review.

Effective health behavior change has been ascribed to rely on the use of behavior change theory. In all, 83% (n=20/24) of studies included in this review reported on the use of behavior change theory. It was beyond the scope of this review to detail how behavior change theory was used to develop the various interventions used in the included studies. This is an area of great interest and could be pursued in future reviews seeking to identify how interventions are constructed, using the methods of Michie and Prestwich [76].

There are a range of studies that looked at synchronous and asynchronous methods in health behavior change interventions in cancer patients and/or survivors, but there is a complete absence of RCTs that compared the differences in the delivery methods within one specific trial. This is where the gap in evidence lies. This systematic review highlights the need for further 3-arm studies comparing both synchronous and asynchronous interventions compared with a standard care group. There should also be an economic evaluation to determine which is also the most cost-effective intervention. Robust reporting of engagement, not only at the initiation of the trial but throughout, is also essential to gain a greater understanding of the complexity of participant engagement in study efficacy and how to replicate this in future implementation of eHealth interventions. Trials of this nature will enable the determination of the most successful method of delivery in terms of effectiveness, acceptability, user engagement, cost-effectiveness, successful behavior change, and ultimately translation into health outcomes.

### Conclusions

This systematic review and meta-analysis provides evidence that behavior change interventions delivered via eHealth, particularly on physical activity and diet modification delivered to cancer patients or survivors, show benefit. There is insufficient evidence to determine whether the specific delivery mode of eHealth (synchronous, asynchronous, or combined) modulates this effectiveness. Three-arm RCTs comparing asynchronous and synchronous delivery modes with a control with robust engagement reporting are required to determine the most successful delivery method for promoting behavior change and ultimately favorable health outcomes.

## Appendix

Multimedia Appendix 1 Preferred reporting items for systematic reviews and meta-analysis checklist.

Multimedia Appendix 2 SCOPUS search strategy.

Multimedia Appendix 3 Descriptive summary.

Multimedia Appendix 4 Behavior change theories and techniques.

## Abbreviations

eHealth: electronic health
mHealth: mobile health
PRISMA: preferred reporting items for systematic reviews and meta-analysis
RCTs: randomized controlled trials
SMD: standardized mean difference

## References

[1] (2016). World Health Organization.

[2] (2011). World Health Organization.

[3] (2019). International Telecommunication Union (ITU).

[4] (2018). Organisation for Economic Co-Operation and Development.

[5] Budman SH, Portnoy D, Villapiano AJ (2003). How to get technological innovation used in behavioral health care: build it and they still might not come. Psychotherapy.

[6] Griffiths F, Lindenmeyer A, Powell J, Lowe P, Thorogood M (2006). Why are health care interventions delivered over the internet? A systematic review of the published literature. J Med Internet Res.

[7] Portnoy DB, Scott-Sheldon LA, Johnson BT, Carey MP (2008). Computer-delivered interventions for health promotion and behavioral risk reduction: a meta-analysis of 75 randomized controlled trials, 1988-2007. Prev Med.

[8] Wantland DJ, Portillo CJ, Holzemer WL, Slaughter R, McGhee EM (2004). The effectiveness of web-based vs non-web-based interventions: a meta-analysis of behavioral change outcomes. J Med Internet Res.

[9] Michie S, van Stralen MM, West R (2011). The behaviour change wheel: a new method for characterising and designing behaviour change interventions. Implement Sci.

[10] Webb TL, Joseph J, Yardley L, Michie S (2010). Using the internet to promote health behavior change: a systematic review and meta-analysis of the impact of theoretical basis, use of behavior change techniques, and mode of delivery on efficacy. J Med Internet Res.

[11] Murray E (2012). Web-based interventions for behavior change and self-management: potential, pitfalls, and progress. Med 2 0.

[12] (2017). International Telecommunication Union (ICT).

[13] Halwas N, Griebel L, Huebner J (2017). eHealth literacy, internet and ehealth service usage: a survey among cancer patients and their relatives. J Cancer Res Clin Oncol.

[14] Hsu J, Huang J, Kinsman J, Fireman B, Miller R, Selby J, Ortiz E (2005). Use of e-health services between 1999 and 2002: a growing digital divide. J Am Med Inform Assoc.

[15] (2018). World Health Organisation.

[16] Aziz NM, Rowland JH (2003). Trends and advances in cancer survivorship research: challenge and opportunity. Semin Radiat Oncol.

[17] Petrick JL, Reeve BB, Kucharska-Newton AM, Foraker RE, Platz EA, Stearns SC, Han X, Windham BG, Irwin DE (2014). Functional status declines among cancer survivors: trajectory and contributing factors. J Geriatr Oncol.

[18] Okwuosa TM, Anzevino S, Rao R (2017). Cardiovascular disease in cancer survivors. Postgrad Med J.

[19] Kushi LH, Doyle C, McCullough M, Rock CL, Demark-Wahnefried W, Bandera EV, Gapstur S, Patel AV, Andrews K, Gansler T, American Cancer Society 2010 Nutrition and Physical Activity Guidelines Advisory Committee (2012). American cancer society guidelines on nutrition and physical activity for cancer prevention: reducing the risk of cancer with healthy food choices and physical activity. CA Cancer J Clin.

[20] Doyle C, Kushi LH, Byers T, Courneya KS, Demark-Wahnefried W, Grant B, McTiernan A, Rock CL, Thompson C, Gansler T, Andrews KS, 2006 Nutrition‚ Physical Activity and Cancer Survivorship Advisory Committee, American Cancer Society (2006). Nutrition and physical activity during and after cancer treatment: an American cancer society guide for informed choices. CA Cancer J Clin.

[21] (2013). Cancer Council Australia.

[22] Kiss N, Baguley BJ, Ball K, Daly RM, Fraser SF, Granger CL, Ugalde A (2019). Technology-supported self-guided nutrition and physical activity interventions for adults with cancer: systematic review. JMIR Mhealth Uhealth.

[23] Goode AD, Lawler SP, Brakenridge CL, Reeves MM, Eakin EG (2015). Telephone, print, and web-based interventions for physical activity, diet, and weight control among cancer survivors: a systematic review. J Cancer Surviv.

[24] Kern J (2006). Evaluation of teleconsultation systems. Int J Med Inform.

[25] Verhoeven F, Tanja-Dijkstra K, Nijland N, Eysenbach G, van Gemert-Pijnen L (2010). Asynchronous and synchronous teleconsultation for diabetes care: a systematic literature review. J Diabetes Sci Technol.

[26] Wootton R (2012). Twenty years of telemedicine in chronic disease management-an evidence synthesis. J Telemed Telecare.

[27] Lee K, Hoti K, Hughes JD, Emmerton L (2017). Dr Google is here to stay but health care professionals are still valued: an analysis of health care consumers' internet navigation support preferences. J Med Internet Res.

[28] Graffigna G, Barello S, Bonanomi A, Riva G (2017). Factors affecting patients' online health information-seeking behaviours: the role of the patient health engagement (PHE) model. Patient Educ Couns.

[29] Higgins J, Thomas J, Chandler J, Cumpston M, Li T, Page M, Welch V (2019). Cochrane Training.

[30] Kirkpatrick D, Kirkpatrick J (2005). Evaluating Training Programs: The Four Levels. Third Edition.

[31] Higgins J, Altman D, Sterne J, Higgins J, Green S (2011). Assessing risk of bias in included studies. Cochrane Handbook for Systematic Reviews of Interventions.

[32] Cohen J (1977). Statistical Power Analysis for the Behavioral Sciences.

[33] Lee MK, Yun YH, Park H, Lee ES, Jung KH, Noh D (2014). A web-based self-management exercise and diet intervention for breast cancer survivors: pilot randomized controlled trial. Int J Nurs Stud.

[34] Higgins J, Li T, Deeks J, Higgins JP, Green S (2019). Choosing effect measures and computing estimates of effect. Cochrane Handbook for Systematic Reviews of Interventions.

[35] Eakin EG, Lawler SP, Winkler EA, Hayes SC (2012). A randomized trial of a telephone-delivered exercise intervention for non-urban dwelling women newly diagnosed with breast cancer: exercise for health. Ann Behav Med.

[36] Hawkes AL, Chambers SK, Pakenham KI, Patrao TA, Baade PD, Lynch BM, Aitken JF, Meng X, Courneya KS (2013). Effects of a telephone-delivered multiple health behavior change intervention (CanChange) on health and behavioral outcomes in survivors of colorectal cancer: a randomized controlled trial. J Clin Oncol.

[37] Ligibel JA, Meyerhardt J, Pierce JP, Najita J, Shockro L, Campbell N, Newman VA, Barbier L, Hacker E, Wood M, Marshall J, Paskett E, Shapiro C (2012). Impact of a telephone-based physical activity intervention upon exercise behaviors and fitness in cancer survivors enrolled in a cooperative group setting. Breast Cancer Res Treat.

[38] Freeman MF, Tukey JW (1950). Transformations related to the angular and the square root. Ann Math Statist.

[39] DerSimonian R, Laird N (1986). Meta-analysis in clinical trials. Control Clin Trials.

[40] Wilson EB (1927). Probable inference, the law of succession, and statistical inference. J Am Stat Assoc.

[41] Bowen DJ, Burke W, Hay JL, Meischke H, Harris JN (2015). Effects of web-based intervention on risk reduction behaviors in melanoma survivors. J Cancer Surviv.

[42] Djuric Z, Ellsworth JS, Weldon AL, Ren J, Richardson CR, Resnicow K, Newman LA, Hayes DF, Sen A (2011). A diet and exercise intervention during chemotherapy for breast cancer. Open Obes J.

[43] Mayer DK, Landucci G, Awoyinka L, Atwood AK, Carmack CL, Demark-Wahnefried W, McTavish F, Gustafson DH (2018). SurvivorCHESS to increase physical activity in colon cancer survivors: can we get them moving?. J Cancer Surviv.

[44] Morey MC, Snyder DC, Sloane R, Cohen HJ, Peterson B, Hartman TJ, Miller P, Mitchell DC, Demark-Wahnefried W (2009). Effects of home-based diet and exercise on functional outcomes among older, overweight long-term cancer survivors: RENEW: a randomized controlled trial. J Am Med Assoc.

[45] Bantum EO, Albright CL, White KK, Berenberg JL, Layi G, Ritter PL, Laurent D, Plant K, Lorig K (2014). Surviving and thriving with cancer using a web-based health behavior change intervention: randomized controlled trial. J Med Internet Res.

[46] Parsons JK, Newman VA, Mohler JL, Pierce JP, Flatt S, Marshall J (2008). Dietary modification in patients with prostate cancer on active surveillance: a randomized, multicentre feasibility study. BJU Int.

[47] Parsons JK, Pierce JP, Natarajan L, Newman VA, Barbier L, Mohler J, Rock CL, Heath DD, Guru K, Jameson MB, Li H, Mirheydar H, Holmes MA, Marshall J (2013). A randomized pilot trial of dietary modification for the chemoprevention of noninvasive bladder cancer: the dietary intervention in bladder cancer study. Cancer Prev Res (Phila).

[48] Pinto B, Dunsiger S, Stein K (2017). Does a peer-led exercise intervention affect sedentary behavior among breast cancer survivors?. Psychooncology.

[49] Porter LS, Gao X, Lyna P, Kraus W, Olsen M, Patterson E, Puleo B, Pollak KI (2018). Pilot randomized trial of a couple-based physical activity videoconference intervention for sedentary cancer survivors. Health Psychol.

[50] Rabin C, Dunsiger S, Ness KK, Marcus BH (2011). Internet-based physical activity intervention targeting young adult cancer survivors. J Adolesc Young Adult Oncol.

[51] Hatchett A, Hallam JS, Ford MA (2013). Evaluation of a social cognitive theory-based email intervention designed to influence the physical activity of survivors of breast cancer. Psychooncology.

[52] Emmons KM, Puleo E, Sprunck-Harrild K, Ford J, Ostroff JS, Hodgson D, Greenberg M, Diller L, de Moor J, Tyc V (2013). Partnership for health-2, a web-based versus print smoking cessation intervention for childhood and young adult cancer survivors: randomized comparative effectiveness study. J Med Internet Res.

[53] Dieng M, Butow PN, Costa DS, Morton RL, Menzies SW, Mireskandari S, Tesson S, Mann GJ, Cust AE, Kasparian NA (2016). Psychoeducational intervention to reduce fear of cancer recurrence in people at high risk of developing another primary melanoma: results of a randomized controlled trial. J Clin Oncol.

[54] Kim SH, Shin MS, Lee HS, Lee ES, Ro JS, Kang HS, Kim SW, Lee WH, Kim HS, Kim CJ, Kim J, Yun YH (2011). Randomized pilot test of a simultaneous stage-matched exercise and diet intervention for breast cancer survivors. Oncol Nurs Forum.

[55] Yun YH, Lee KS, Kim Y, Park SY, Lee ES, Noh D, Kim S, Oh JH, Jung SY, Chung K, Lee YJ, Jeong S, Park KJ, Shim YM, Zo JI, Park JW, Kim YA, Shon EJ, Park S (2012). Web-based tailored education program for disease-free cancer survivors with cancer-related fatigue: a randomized controlled trial. J Clin Oncol.

[56] Kanera IM, Bolman CA, Willems RA, Mesters I, Lechner L (2016). Lifestyle-related effects of the web-based Kanker Nazorg Wijzer (cancer aftercare guide) intervention for cancer survivors: a randomized controlled trial. J Cancer Surviv.

[57] Ormel HL, van der Schoot GG, Westerink ND, Sluiter WJ, Gietema JA, Walenkamp AM (2018). Self-monitoring physical activity with a smartphone application in cancer patients: a randomized feasibility study (SMART-trial). Support Care Cancer.

[58] Golsteijn RH, Bolman C, Volders E, Peels DA, de Vries H, Lechner L (2018). Short-term efficacy of a computer-tailored physical activity intervention for prostate and colorectal cancer patients and survivors: a randomized controlled trial. Int J Behav Nutr Phys Act.

[59] Forbes CC, Blanchard CM, Mummery WK, Courneya KS (2015). Feasibility and preliminary efficacy of an online intervention to increase physical activity in Nova Scotian cancer survivors: a randomized controlled trial. JMIR Cancer.

[60] Villaron C, Cury F, Eisinger F, Cappiello M, Marqueste T (2018). Telehealth applied to physical activity during cancer treatment: a feasibility, acceptability, and randomized pilot study. Support Care Cancer.

[61] Pinto BM, Stein K, Dunsiger S (2015). Peers promoting physical activity among breast cancer survivors: a randomized controlled trial. Health Psychol.

[62] Michie S, Richardson M, Johnston M, Abraham C, Francis J, Hardeman W, Eccles MP, Cane J, Wood CE (2013). The behavior change technique taxonomy (v1) of 93 hierarchically clustered techniques: building an international consensus for the reporting of behavior change interventions. Ann Behav Med.

[63] Abraham C, Michie S (2008). A taxonomy of behavior change techniques used in interventions. Health Psychol.

[64] Cox M, Basen-Engquist K, Carmack CL, Blalock J, Li Y, Murray J, Pisters L, Rodriguez-Bigas M, Song J, Cox-Martin E, Demark-Wahnefried W (2017). Comparison of internet and telephone interventions for weight loss among cancer survivors: randomized controlled trial and feasibility study. JMIR Cancer.

[65] Davies CA, Spence JC, Vandelanotte C, Caperchione CM, Mummery WK (2012). Meta-analysis of internet-delivered interventions to increase physical activity levels. Int J Behav Nutr Phys Act.

[66] Eysenbach G (2005). The law of attrition. J Med Internet Res.

[67] Kelders SM, Kok RN, Ossebaard HC, van Gemert-Pijnen JE (2012). Persuasive system design does matter: a systematic review of adherence to web-based interventions. J Med Internet Res.

[68] Short C, Rebar A, Vandelanotte C (2015). Designing engaging online behaviour change interventions: a proposed model of user engagement. Eur Psychol.

[69] Wanner M, Martin-Diener E, Bauer G, Braun-Fahrländer C, Martin BW (2010). Comparison of trial participants and open access users of a web-based physical activity intervention regarding adherence, attrition, and repeated participation. J Med Internet Res.

[70] Yardley L, Spring BJ, Riper H, Morrison LG, Crane DH, Curtis K, Merchant GC, Naughton F, Blandford A (2016). Understanding and promoting effective engagement with digital behavior change interventions. Am J Prev Med.

[71] Perski O, Blandford A, West R, Michie S (2017). Conceptualising engagement with digital behaviour change interventions: a systematic review using principles from critical interpretive synthesis. Transl Behav Med.

[72] Eakin EG, Lawler SP, Vandelanotte C, Owen N (2007). Telephone interventions for physical activity and dietary behavior change: a systematic review. Am J Prev Med.

[73] Cornwell P, Dicks B, Fleming J, Haines TP, Olson S (2012). Care and support needs of patients and carers early post-discharge following treatment for non-malignant brain tumour: establishing a new reality. Support Care Cancer.

[74] Free C, Phillips G, Galli L, Watson L, Felix L, Edwards P, Patel V, Haines A (2013). The effectiveness of mobile-health technology-based health behaviour change or disease management interventions for health care consumers: a systematic review. PLoS Med.

[75] Rosenman R, Tennekoon V, Hill LG (2011). Measuring bias in self-reported data. Int J Behav Healthc Res.

[76] Michie S, Prestwich A (2010). Are interventions theory-based? Development of a theory coding scheme. Health Psychol.

