# Supplementary figures and images for: Impact of the Method of Delivering Electronic Health Behavior Change Interventions in Survivors of Cancer on Engagement, Health Behaviors, and Health Outcomes: Systematic Review and Meta-Analysis
Source: J Med Internet Res. 2020 Jun 23;22(6):e16112. doi: 10.2196/16112 (PMC7381039; doi:10.2196/16112)

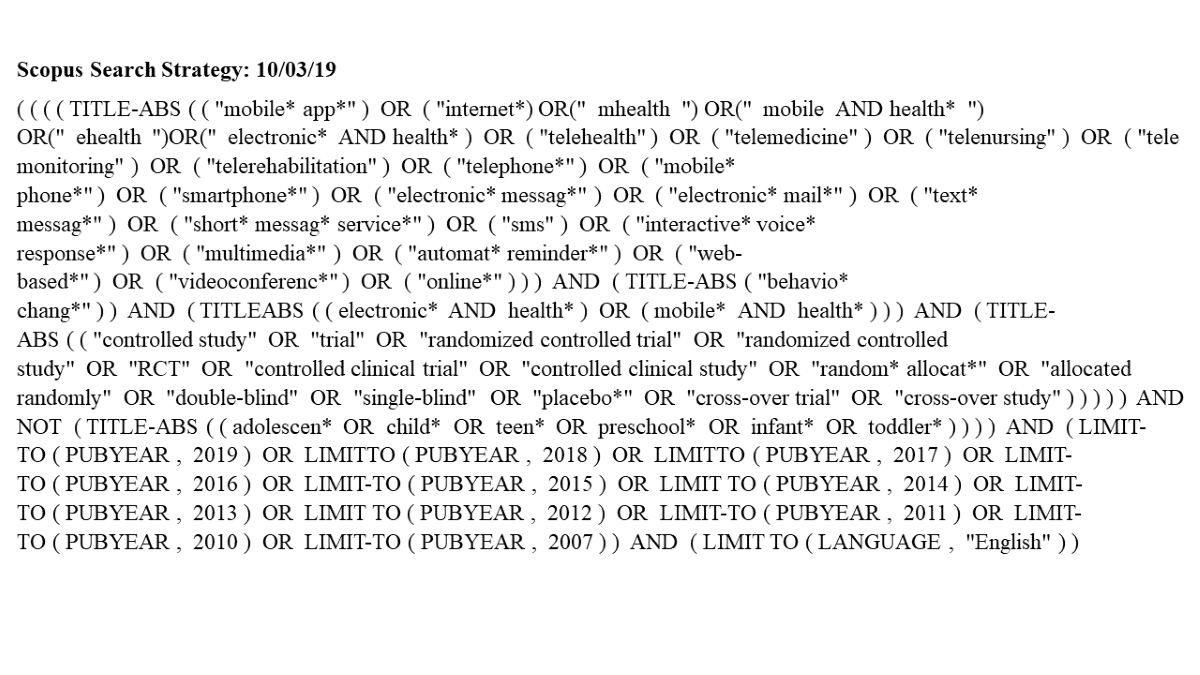

Supplement: Multimedia Appendix 2 [file jmir_v22i6e16112_app2.png]
