# Supplementary material for: Impact of the Method of Delivering Electronic Health Behavior Change Interventions in Survivors of Cancer on Engagement, Health Behaviors, and Health Outcomes: Systematic Review and Meta-Analysis
Source: J Med Internet Res. 2020 Jun 23;22(6):e16112. doi: 10.2196/16112 (PMC7381039; doi:10.2196/16112)
Supplement: Multimedia Appendix 3 [file jmir_v22i6e16112_app3.docx]

| First author, country | Study design | Delivery method | Participants | Interventions | Other | Intervention participation rate | Control | Outcomes | Follow-up after the end of active intervention |
| --- | --- | --- | --- | --- | --- | --- | --- | --- | --- |
| O'Carroll-Bantum et al, United States [45] | RCT^a^   - (1) Web-based intervention - (2) Wait list control | Asynchronous: web-based | Completion of primary cancer treatment 4 weeks to 5 years  N=352, consent rate=56.5%, retention rate=86% | Intervention: 6 week web-based patient education workshop of 22 topics, including fatigue management, exercise, healthy eating, and stress management. | Other: Web-based training provided for website and goal setting | 67% accessed all sessions  Mean number of sessions 5.3 (SD 1.28) | Wait list: no information or materials provided | Measured: baseline and 6 months  Primary outcomes: fruit and vegetable intake, PA^b^, depression, fatigue, and insomnia | No |
| Eakin et al, Australia [35] | RCT   - (1) Telephone intervention - (2) Usual care | Synchronous: telephone | Women with newly diagnosed breast cancer who are >6 weeks post surgery  N=143, consent rate=40%, retention rate=96% | Intervention: 16 telephone calls over 8 months delivered by an exercise physiologist targeting aerobic-based exercise 4 days per week for 45 min and 2 sessions resistance training per week. | Other: Workbook and exercise tracker | Median participation was 14/16 telephone calls  79% completed the majority (≥75%) of calls. | Usual care: no intervention contact. Workbook and exercise tracker sent at the end of intervention period | Measured: baseline, 6, and 12 months.  Primary outcomes: aerobic exercise and resistance training.  Secondary outcomes: QoL^c^, fatigue, anxiety, upper body function, and self-efficacy to exercise. | 17 weeks |
| Hawkes et al, Australia [36] | RCT   - (1) Telephone intervention - (2) Usual care | Synchronous: telephone | Colorectal cancer survivors N=410, consent rate=46%, retention rate=78.5% | Intervention: 11 telephone sessions over 6 months focused on PA, weight management, diet, alcohol, and smoking | Other: Participant handbook, regular motivational postcard prompts, a pedometer, and quarterly study newsletter. | 72% received all 11 telephone calls.  Median=10. | Usual care: Cancer Council Australia brochures and quarterly study newsletter | Measured: baseline, 6, and 12 months  Primary outcomes: Physical activity, HRQOL^d^, cancer-related fatigue  Secondary outcomes: BMI, diet and alcohol intake, smoking | 26 weeks |
| Bowen et al, United States [41] | RCT   - (1) Web-based intervention - (2) Control group | Asynchronous: web-based | Melanoma survivors and their family members N=311, consent rate not reported, retention rate=89% | Intervention: Specifically designed website with 12-month access Content included: individualized chronic disease risk, risk reduction, sun exposure prevention, self-screening, chat room, and family-based interactive section. | Other: Email prompts sent every 3 months | 96% participants visited the website, total 1.9 times during intervention period. | Not described | Measured: baseline and 12 months  Primary outcomes: Skin self-examination, sun protection behaviors, and provider screening.  Secondary outcomes: perceived risk of future cancer and cancer worry | No |
| Morey et al, United States [44] | RCT   - (1) Telephone intervention - (2) Wait list control | Synchronous: telephone | Overweight or obese, older, long-term colorectal, breast and prostate cancer survivors  N=641, consent rate=30%, retention rate=87.1% | Intervention: 12-month home-based diet-exercise intervention with mailed print materials and telephone counselling in line with America Cancer Society and the US Dietary Guidelines  Content included: strength training, endurance exercise, fruit and vegetable consumption, weight loss, and reduced saturated fat intake. | Other: Personalized workbook, pedometer, resistance exercise bands, instruction-based exercise poster, Portion Doctor tableware, and personalized record logs for self-monitoring. | Average of 62% of the 15 telephone counselling sessions. | Wait list | Measured: baseline and 12 months  Primary outcomes: change in functional status  Secondary outcomes: PA, diet, weight loss, and QoL | No |
| Djuric et al, United States [42] | RCT   - (1) Telephone intervention - (2) Wait list control | Synchronous: telephone | Women undergoing chemotherapy for stage I to IIIA breast cancer N=40, consent rate=55%, retention rate=75% | Intervention: 12-month telephone counselling (19 total calls) intervention with print materials. Content included: goals related to fruit, vegetable and fat consumption, and PA | Other: All participants received pedometers, study newsletter. Intervention received food and exercise log, fat gram counter, and example menus. | 17/19 proposed calls were completed. | My Pyramid plan, Dietary Guidelines for Americans, Choices for Good Health and Cooking Smart brochures, pedometers, and study newsletters. | Measured: baseline, 6, and 12 months.  Primary outcome: changes in body fat and weight  Secondary outcomes: psychosocial well-being and compliance to PA and diet goals | No |
| Forbes et al, Canada [59] | RCT   - (1) Web-based intervention - (2) Usual care | Asynchronous: web-based | Nova Scotian colorectal, breast, and prostate cancer survivors  N=95, consent rate=unclear, retention rate=88% | Intervention: 9 week (1 per week) web-based exercise modules. Content included: goal setting, exercise myths, exercise safety, benefits exercise, social support, and barriers and strategies for exercise, relapse tips. | Other: Accelerometer via UWALK website logged step counts. Email updates of new content. Brief summary of previous week. | 67% viewed modules once. 26% modules were completed.  Average log-ins 10.3. | Usual care: maintain usual routine over next 9 weeks. | Measured: baseline and 9 weeks  Primary outcomes: website usage, engagement, adherence, and acceptability  Secondary outcome: PA | No |
| Kaneraet al, the Netherlands [56] | RCT   - (1) Web-based intervention - (2) Usual care | Asynchronous: web-based | Cancer survivors between 4 weeks and 56 weeks after treatment N=462, consent rate=36%, retention rate=88.5% | Intervention: 6 month, 8 module Cancer Aftercare Guide intervention delivered via web-based content. Included: Problem ID, goal setting, written and video content for nutrition, exercise, smoking, fatigue, anxiety, depression, relational problems, and return to work. | Other: email reminders, monthly news items with additional information. | Average 2.23 (SD 1.58) modules.  25% followed PA module.  62% followed diet module. | Usual care: face-to-face after care | Measured: baseline, 6, and 12 months  Primary outcomes: PA, dietary behavior  Secondary outcome: smoking behavior | 26 weeks |
| Kim et al, South Korea [54] | RCT   - (1) Telephone intervention - (2) Wait list control | Synchronous: telephone | Breast cancer survivors  N=45, consent rate=19.7%, retention rate=80% | Intervention: 12-week stage of change-matched diet and exercise intervention delivered via telephone counselling. Individualized prescription for PA and balanced diet based on Korean Nutrition Society guidelines. | Other: new workbook at each new stage of change, portable heart rate monitor. | 100% completed telephone counselling each week.  94% adherence to exercise goals and 91% adherence to diet goals. | Not described | Measured: baseline and 12 weeks.  Primary outcome(s): intervention feasibility, stage of motivational readiness, PA, diet quality, functioning and global QoL, fatigue, anxiety, and depression | No |
| Ligibel et al, United States [37] | RCT   - (1) Telephone intervention - (2) Wait list control | Synchronous: telephone | Breast, colon, or rectal cancer survivors 2 to 36 months post treatment completion  N=121. consent rate=51%, retention rate=82% | Intervention: 16 week telephone exercise intervention, 10 to 11 calls. Target exercise 180 min moderate intensity exercise PA per week. | Other: pedometer, participant workbook. | Median 9 out of planned 10 to 11 telephone calls. | Routine care. Offered telephone consultation with exercise trainer at the end of the control period. | Measured: baseline and 16 weeks.  Primary outcome: change in minutes of PA.  Secondary outcomes: changes in physical functioning, fitness, anthropometric measures, fatigue, and QoL. | No |
| Mayer et al, United States [43] | RCT   - (1) Web-based intervention - (2) Control | Asynchronous: web-based | Stage I to III colon cancer survivors, completed treatment, 6 weeks postoperative and 12 months postdiagnosis  N=284, consent rate=61%, retention rate=80% | Intervention: 6 month PA intervention delivered via mobile app, smartphones included. Included information, skill development, and support. | Other: SMS notifications. Facing Forward: Life After Cancer Treatment, booklet, National Coalition for Cancer Survivorship’s Cancer Survival Toolbox and pedometer. | 93.8% accessed the system.  Mean use 55.3 (SD 50.0) out of 180 possible days. | Facing Forward: Life After Cancer Treatment, booklet, National Coalition for Cancer Survivorship’s Cancer Survival Toolbox and pedometer. | Measured: baseline, 3, 6, and 9 months.  Primary outcome: PA  Secondary outcomes: QoL and distress | 12 weeks |
| Lee et al, South Korea [33] | RCT   - (1) Web-based intervention - (2) Active control | Asynchronous: web-based | Stage 0 to III breast cancer survivors within 12 months of curative surgery and/or treatment  N=59, consent rate=34%, retention rate=97% | Intervention: 12 week web-based intervention. Content included: 5 modules based on stage of change for exercise and diet behaviors, barriers to sustainability, and weight management. | Other: Training on use of web-based program, no content was discussed. SMS prompted participants to access web content twice per week. | 89% participated consistently | 50 page educational booklet on diet and exercise | Measured: baseline and 12 weeks.  Primary outcomes: exercise and dietary behaviors, dietary quality.  Secondary outcomes: HRQOL, anxiety, depression, fatigue, motivational readiness, and self-efficacy. | No |
| Ormel et al, the Netherlands [57] | RCT   - (1) Web-based intervention - (2) Usual care | Asynchronous: web-based | Cancer patients having active systemic treatment or survivors  N=32, consent rate=53%, retention rate=97% | Intervention: 12 week PA intervention delivered via mobile app RunKeeper. | Other: brief users guide for RunKeeper use. Troubleshooting was done via telephone or email. | Not reported. | Usual care: informed regular PA is safe, feasible, and enhances recovery and physical health. | Measured: baseline, 6, and 12 weeks.  Primary outcomes: Self-reported PA.  Secondary outcomes: usability and patients experience of RunKeeper app. | No |
| Parsons, United States [46] | RCT   - (1) Telephone intervention - (2) Control | Synchronous: telephone or Skype | Noninvasive urothelial cell carcinoma with at least three years life expectancy  N=48, consent rate: unable to calculate, retention rate=83% | Intervention: 12 calls over 6-month intervention period to guide and support patient to meet target vegetable consumption. | Nil | 83% participants completed dietary recall and 81% completed blood and urine samples. | Control: Dietary Guidelines for Americans | Measured: baseline and 6 months.  Primary outcome: 7 vegetable serves per day with at least two cruciferous vegetables. | No |
| Parsons, United States [47] | RCT   - (1) Telephone intervention - (2) Control | Synchronous: telephone | Active surveillance for prostate cancer  N=43, consent rate: not presented, retention rate=98% | Intervention: 13 structured telephone-based counselling sessions over 6 months to effect dietary change. Target to achieve 7 serves per day vegetables, 2 serves whole grains, and 1 serve of beans/legumes. | Nil | Not reported. | Control: printed materials on standard guidelines recommending 5 servings of vegetables and fruits daily. | Measured: baseline and 6 months.  Primary outcomes: differences in dietary intake and plasma carotenoid concentrations. | No |
| Pinto et al, United States [61] (Secondary outcomes: Pinto et al, United States [48]) | RCT   - (1) Telephone intervention - (2) Control | Synchronous: telephone | Breast cancer survivors  N=76, consent rate=71%, retention rate=92% intervention, 87% control | Intervention: 12 week telephone-based PA intervention to gradually increase PA to 30 min per day of moderate intensity activity. | Other: pedometer, heart rate monitor, logbooks, Reach to Recovery print materials, 12 exercise tip sheets, and PA feedback reports at week 2, 4, 8, and 12. Accelerometer used for data collection. | Not reported. | Control: Weekly phone calls administering weekly symptom questionnaire assessing general health problems. Reach To Recovery print materials. At 24 weeks they were sent exercise tip sheets. | Measured: baseline, 12, and 24 weeks.  Primary outcome: minutes of sedentary time per week.  Secondary outcomes: fatigue and QoL. | 12 weeks |
| Porter et al, United States [49] | RCT   - (1) Videoconferencing intervention - (2) Wait list control | Synchronous: videoconference | Sedentary cancer survivors and their partners  N=20, consent rate: unable to calculate, retention rate=92% | Intervention: 4×60 min videoconference sessions over 2 months. Content included: identification of social support strategies, joint decision making for goals and plans to increase PA, barrier identification, and strategies. | Other: loaned tablet computers with 3G internet access and instructed on their use. | 78% completed all 4 sessions. | Wait list control | Measured: baseline and 8 weeks.  Primary outcome: feasibility of recruiting and retaining couples and acceptability of intervention.  Secondary outcomes: survivor and partner PA, partner support for PA, and survivor well-being. | No |
| Yun et al, Korea [55] | RCT   - (1) Web-based intervention - (2) Wait list control | Asynchronous: web-based | Cancer survivors <24 months posttreatment  N=273, consent rate=88%, retention rate=89% | Intervention: 12-week web-based intervention of 7 modules based on National Comprehensive Cancer Network guidelines. Content included: energy conservation, PA, nutrition, sleep hygiene, pain, distress, and fatigue management. | Other: booklet explaining Health Navigation website. Tailored email and short messaging service. | 83.1% participants completed the 12-week course. | Wait list control: personalized letter reminded them of assessment points | Measured: baseline and 12 weeks.  Primary outcome: cancer-related fatigue.  Secondary outcomes: health-related QoL, energy conservation, PA, nutrition, sleep hygiene, pain, anxiety, depression, and distress. | No |
| Rabin et al, United States [50] | RCT   - (1) Web-based intervention - (2) Control | Asynchronous: web-based | Young adult cancer survivors within 10 years diagnosis  N=18, consent rate=72%, retention rate=94% | Intervention: 12 weeks access to website Step into Motion. Stage-matched, tailored PA intervention with weekly goal setting, feedback, information, and resources. | Other: initial training to website, weekly emails monitoring physical symptoms. | 14.75 (SD 8.46) average log-ins to website | Control: 3 publicly available websites—I’m Too Young For This!, Planet Cancer, and Cancer Care. No information about PA included. | Measured: baseline and 12 weeks.  Primary outcomes: feasibility and acceptability.  Secondary outcomes: PA, mood, and fatigue. | No |
| Dieng et al, Australia [53] | RCT   - (1) Telephone intervention - (2) Control | Synchronous: telephone | Melanoma survivors  N=164, consent rate=58%, retention rate=83% | Intervention: 3 psychotherapeutic telephone calls facilitated by psychologist to develop effective emotional and behavioral coping strategies. | Other: Psycho-education booklet—Melanoma: Questions and Answers | 96% completed all 3 sessions.  Total of 1.66 hours telephone counselling (SD 0.93).  88% intervention fidelity. | Usual care: usual dermatologic appointments and a hard copy of the Cancer Council Australia booklet ”Understanding Melanoma.” | Measured: baseline, 1, 6, and 12 months.  Primary outcome: fear of new or recurrent melanoma.  Secondary outcomes: anxiety, stress, depression, melanoma knowledge, health behaviors, satisfaction with melanoma care, unmet needs, and HRQoL | 26 weeks |
| Golsteijn et al, the Netherlands [58] | RCT   - (1) Web-based intervention - (2) Wait list control | Asynchronous: web-based | Colorectal or prostate cancer patients and survivors  N=478, consent rate: unable to calculate, retention rate=86% | Intervention: A 4-month computer-tailored PA advice via the OncoActiv website at 3 time points both web-based on a secured website and on paper (by mail). | Other: pedometer, interactive website content, the option to consult a physical therapist, and additional information. | Not reported. | Wait list control | Measured: Baseline, 3, and 6 months (12 months)  Primary outcome: PA behavior  Secondary outcome:  fatigue, distress, and HRQoL | 10 months |
| Hatchet et al, United States [51] | RCT   - (1) Email intervention - (2) Control | Asynchronous: email | Breast cancer survivors  N=74, consent rate=43%, retention rate=87% | Intervention: email messages targeting PA. Weekly emails for 6 weeks, fortnightly emails for remaining weeks. Access to electronic counsellor (exercise physiologist). | Nil | Not reported | Control: offered intervention at the end of the study | Measured: baseline, 6, and 12 weeks  Primary outcome: PA  Secondary outcome: self-regulation, outcome expectancy value, exercise self-efficacy, and exercise role identity (outcomes not reported) | No |
| Villaron et al, France [60] | RCT   - (1) Telehealth and SMS message - (2) Control | Combined: telephone and SMS message | Any cancer type receiving chemotherapy or systemic treatment  N=60, consent rate=60%, retention rate=72% | Intervention: 8 weeks of advice on increasing PA and weekly SMS text messages to encourage PA. | Other: pedometer, paper-based PA recommendation guide | 65% web-based questionnaires completed.  Considered 71% participated convincingly | Control: pedometer | Measured: Weeks 1-8  Primary outcome: PA  Secondary outcome: Fatigue and QoL | No |
| Emmons et al, United States [52] | RCT   - (1) Web and online forum - (2) Usual care | Combined: web-based and peer-moderated discussion forum | Survivors of childhood or young adult cancers  N=374, consent rate=12%, retention rate=88% | Intervention: 6 month Partnership for Health-2 self-directed–web-based intervention. 7 sessions tailored to stage of readiness to quit smoking | Other: free pharmacotherapy (smoking cessation aids); online peer counsellor discussion forum, biweekly newsletter, and reminder emails. | 58% logged on once (58% used print materials)  14% requested pharmacotherapy | Usual care: paper-based manual, free pharmacotherapy (smoking cessation aids) | Measured: baseline, 6 months, and 15 months  Primary outcome: smoking cessation  Secondary outcome: Smoking quit attempts, readiness to change, intervention use, satisfaction, and access to web-based intervention | 9 months |

^a^RCT: randomized controlled trial.

^b^PA: physical activity.

^c^QoL: quality of life.

^d^HRQoL: health-related quality of life.
