# Supplementary material for: Impact of the Method of Delivering Electronic Health Behavior Change Interventions in Survivors of Cancer on Engagement, Health Behaviors, and Health Outcomes: Systematic Review and Meta-Analysis
Source: J Med Internet Res. 2020 Jun 23;22(6):e16112. doi: 10.2196/16112 (PMC7381039; doi:10.2196/16112)
Supplement: Multimedia Appendix 4 [file jmir_v22i6e16112_app4.docx]

| First author, country | Behavior change theory | Behavior change technique(s) |
| --- | --- | --- |
| O'Carroll-Bantum et al, United States [45] | None described | - 1.1 Goal setting (behavior) - 1.2 Problem solving - 1.4 Action planning - 2.2 Feedback on behavior - 2.3 Self-monitoring of behavior - 3.1 Social support (unspecified) - 5.1 Information on health consequences - 6.2 Social comparison - 11.2 Reduce negative emotions |
| Eakin et al, Australia [35] | Social cognitive theory | - 1.1 Goal setting (behavior) - 1.2 Problem solving - 1.3 Goal setting (outcome) - 1.4 Action planning - 1.5 Review behavior goals - 1.6 Review outcome goals - 2.2 Feedback on behavior - 2.3 Self-monitoring of behavior - 2.6 Biofeedback - 2.7 Feedback on outcomes of behavior - 4.1 Instruction on how to perform behavior - 5.1 Information on health consequences - 8.1 Graded tasks - 15.3 Focus on past success |
| Hawkes et al, Australia [36] | Acceptance commitment therapy | - 1.1 Goal setting (behavior) - 1.2 Problem solving - 1.4 Action planning - 1.5 Review of behavior goals - 2.2 Feedback on behavior - 2.3 Self-monitoring of behavior - 2.6 Biofeedback - 5.1 Information on health consequences - 7.1 Prompts/cues |
| Bowen et al, United States [41] | None described | - 4.1 Instruction on how to perform behavior - 5.1 Information on health consequences - 6.1 Demonstration of behavior |
| Morey et al, United States [44] | Social cognitive theory and transtheoretical model | - 1.1 Goal setting (behavior) - 1.2 Problem solving - 1.3 Goal setting (outcome) - 1.5 Review behavior goal(s) - 1.7 Review outcomes goal(s) - 2.2 Feedback on behavior - 2.3 Self-monitoring of behavior - 2.4 Self-monitoring of outcome(s) of behavior - 2.6 Biofeedback - 2.7 Feedback on outcome(s) of behavior - 4.1 Instruction on how to perform a behavior - 5.1 Information about health consequences - 6.1 Demonstration of the behavior - 8.7 Graded tasks - 9.1 Credible source - 12.6 Body changes - 15.3 Focus on past success |
| Djuric et al, United States [42] | Social cognitive theory and motivational interviewing | - 1.1 Goal setting (behavior) - 1.5 Review behavior goals - 2.2 Feedback in behavior - 2.3 Self-monitoring of behavior - 2.6 Biofeedback - 5.1 Information on health consequences |
| Forbes et al, Canada [59] | Transtheoretical model | - 1.1 Goal setting (behavior) - 1.2 Problem solving - 1.4 Action planning - 1.6 Discrepancy between current behavior and goals - 2.2 Feedback on behaviors - 2.3 Self-monitoring of behaviors - 3.1 Social support (unspecified) - 4.1 Instructions on how to perform behavior - 5.1 Information about health consequences - 6.2 Social comparison - 9.1 Credible source |
| Kanera et al, the Netherlands [56] | Integrated model for change (I-change model), self-regulation theory, and problem-solving therapy | - 1.1 Goal setting (behavior) - 1.2 Problem solving - 1.3 Goal setting (outcome) - 1.4 Action planning - 1.6 Discrepancy between current behavior and goals - 2.2 Feedback on behaviors - 2.3 Self-monitoring of behaviors - 4.1 Instructions on how to perform behavior - 5.1 Information about health consequences - 6.2 Social comparison - 9.1 Credible source - 9.2 Pros and cons |
| Kim et al, South Korea [54] | Transtheoretical model of change | - 1.1 Goal setting (behavior) - 1.2 Problem Solving - 1.3 Goal setting (outcome) - 1.4 Action planning - 1.5 Review behavior - 1.6 Review outcome(s) of behavior - 2.2 Feedback on behavior - 2.3 Self-monitoring of behavior - 2.4 Self-monitoring of outcome(s) of behavior - 2.6 Biofeedback - 2.7 Feedback on outcome(s) of behavior 3.1 Social support (unspecified) - 8.2 Behavior substitution - 9.3 Comparative imagining of future outcomes - 10.9 Self reward - 12.3 Avoidance/reducing exposure to cues for the behavior - 15.3 Focus on past success |
| Ligibel et al, United States [37] | Social cognitive theory | - 1.1 Goal setting (behavior) - 1.2 Problem solving - 1.5 Review of behavioral goals - 2.2 Feedback on behavior - 2.3 Self-monitoring of behavior - 2.6 Biofeedback - 5.1 Information on health consequences |
| Mayer et al, United States [43] | Self-determination theory | - 1.1 Goal setting (behavior) - 2.3 Self-monitoring of behavior - 2.6 Biofeedback - 3.1 Social support (unspecified) - 4.1 Instructions on how to perform a behavior - 5.1 Information on health consequences - 9.1 Credible source |
| Lee et al, South Korea [33] | Transtheoretical model | - 1.1 Goal setting (behavior) - 1.2 Problem solving - 1.4 Action planning - 1.6 Discrepancy between current behavior and goal - 2.2 Feedback on behavior - 2.3 Self-monitoring of behavior - 4.1 Instruction on how to perform the behavior - 5.1 Information about health consequences - 9.1 Credible source |
| Ormel et al, the Netherlands [57] | None described | - 2.3 Self-monitoring of behavior - 7.1 Prompts/Cues |
| Parsons et al, United States [46] | Social cognitive theory | - 1.1 Goal setting (behavior) - 4.1 Instruction on how to perform behavior |
| Parsons et al, United States [47] | Social cognitive theory | - 1.1 Goal setting (behavior) - 1.2 Problem solving - 1.4 Action planning - 2.3 Self-monitoring of behavior - 4.1 Instruction on how to perform behavior - 5.1 Information about health consequences - 7.1 Prompts/cues |
| Pinto et al, United States [61] (Secondary outcomes: Pinto et al, United States [48]) | Transtheoretical model and social cognitive theory | - 1.1 Goal setting (behavior) - 1.2 Problem solving - 1.4 Action planning - 1.5 Review of behavior goals - 1.6 Discrepancy between current behavior and goal - 2.3 Feedback on behavior - 2.4 Self-monitoring of behavior - 2.6 Biofeedback - 4.1 Instruction on how to perform behavior - 6.1 Demonstration of behavior - 8.7 Graded tasks |
| Porter et al, United States [49] | Interdependence theory | - 1.1 Goal setting (behavior) - 1.2 Problem solving - 1.4 Action Planning - 2.4 Self-monitoring of behavior - 3.1 Social support (unspecified) - 7.1 Prompts/cues |
| Yun et al, Korea [55] | Transtheoretical model, social cognitive theory, and cognitive behavioral therapy | - 1.4 Action Planning - 2.1 Monitoring outcomes of behavior by others without feedback - 2.2 Feedback on behavior - 3.1 Social support (unspecified) - 4.1 Instructions on how to perform a behavior - 5.1 Information about health consequences |
| Rabin et al, United States [50] | Transtheoretical model and social cognitive theory | - 1.1 Goal setting (behavior) - 2.3 Feedback on behavior - 2.4 Self-monitoring of behavior - 3.1 Social support (unspecified) - 4.1 Instructions on how to perform a behavior - 5.1 Information about health consequences |
| Dieng et al, Australia [53] | Brief psycho-dynamically oriented psychotherapy | - 1.1 Goal setting (behavior) - 1.2 Problem solving - 1.3 Goal setting (outcome) - 1.4 Action planning - 2.3 Self-monitoring of behavior - 2.4 Self-monitoring of outcomes of behavior - 4.1 Instruction on how to perform a behavior - 5.1 Information about health consequences - 5.4 Monitoring of emotional consequences - 5.6 Information about emotional consequences - 6.1 Demonstration of behavior - 9. Credible source - 11.2 Reduce negative emotions |
| Golsteijn et al, the Netherlands [58] | I- change model, social cognitive theory, transtheoretical model, health belief model, goal setting theories, health action process approach, theories of self-regulation, and precaution adoption process model | - 1.1 Goal setting (behavior) - 1.2 Problem solving - 1.3 Action planning - 1.5 Review of behavior goals - 1.6 Discrepancy between current behavior and goal - 2.2 Feedback on behavior - 2.3 Self-monitoring of behavior - 2.6 Biofeedback - 4.1 Instruction on how to perform behavior - 5.1 Information about health consequences - 6.1 Demonstration of behavior - 6.2 Social comparison - 9.1 Credible source |
| Hatchett et al, United States [51] | Social cognitive theory | - 1.1 Goal setting (behavior) - 1.2 Problem solving - 2.3 Self-monitoring of behavior - 3.1 Social support (unspecified) - 9.1 Credible source - 13.5 Identity associated with changed behavior - 15.1 Verbal persuasion about capability |
| Villaron et al, France [60] | None described | - 1.1 Goal setting (behavior) - 2.6 Biofeedback - 6.1 Demonstration of the behavior |
| Emmons et al, United States [52] | Social cognitive theory, the transtheoretical model, and precaution adoption process model | - 1.2 Problem solving - 1.3 Action planning - 3.1 Social support (unspecified) - 5.1 Information about health consequences - 7.1 Prompts/cues - 11.1 Pharmacological support |
